# Ultrasound-responsive nanoplatforms for cancer immunotherapy: engineering spatiotemporal immune activation in the tumor microenvironment

**DOI:** 10.3389/fimmu.2026.1988247

**Published:** 2026-10-19

**Authors:** Qiulan Zhang, Tao Wei, Zhenhao Lan, Guojin Lin, Meihong Xu, Xiaoyan Li, Yanni Bao

**Affiliations:** 1 Department of Ultrasound, The Second Affiliated Hospital of Xiamen Medical College, Xiamen, Fujian, China; 2 Department of Traditional Chinese Medicine, The Second Affiliated Hospital of Xiamen Medical College, Xiamen, Fujian, China; 3 Department of Internal Medicine, The Second Affiliated Hospital of Xiamen Medical College, Xiamen, Fujian, China; 4 Department of Gynecology, The Second Affiliated Hospital of Xiamen Medical College, Xiamen, Fujian, China

**Keywords:** cancer immunotherapy, sonodynamic therapy, STING activation, tumor microenvironment, ultrasound-responsive nanoplatforms

## Abstract

Immune checkpoint blockade has reshaped cancer therapy, but durable responses remain limited by insufficient antigen release, poor T-cell infiltration, immunosuppressive tumor microenvironments, and systemic immune-related toxicity. Ultrasound-responsive nanoplatforms provide a spatiotemporally controllable strategy to overcome these barriers by exploiting deep tissue penetration, focal activation, and repeatable external triggering. In this review, we summarize recent experimental advances in sonosensitizer-based and ultrasound-triggered nanoplatforms for cancer immunotherapy. These systems integrate sonodynamic immunogenic cell death, pyroptosis, ferroptosis, necroptosis, cGAS-STING activation, immune checkpoint blockade, mRNA delivery, gas therapy, and tumor microenvironment remodeling to convert immune-cold tumors into immune-responsive lesions. We further discuss cancer type-specific applications in hepatocellular carcinoma, pancreatic cancer, glioblastoma, triple-negative breast cancer, colorectal cancer, bladder cancer, and other solid tumors, emphasizing how ultrasound-responsive designs can be matched to distinct anatomical and immunological barriers. Finally, we highlight key translational challenges, including long-term biosafety, controllable immune activation, ultrasound parameter standardization, scalable manufacturing, and clinically compatible combination strategies. Ultrasound-responsive nanoplatforms are emerging not merely as drug carriers, but as programmable immune-engineering systems capable of coordinating when, where, and how antitumor immunity is activated within the tumor microenvironment.

## Highlights

- Ultrasound enables spatially and temporally controlled immune activation in deep tumors.
- Sono-immunotherapy integrates ICD, STING activation, ICB, mRNA delivery, and TME remodeling.
- Clinical translation requires safer materials, standardized ultrasound dosing, and rational combinations.

## 1 Introduction

Immune checkpoint blockade (ICB), represented by antibodies targeting programmed cell death protein 1 (PD-1), programmed death ligand 1 (PD-L1), and cytotoxic T lymphocyte-associated antigen 4 (CTLA-4), has reshaped the therapeutic landscape of multiple malignancies by releasing inhibitory constraints on antitumor T-cell immunity (1, 2). Durable tumor regression can be achieved in a subset of patients, supporting the concept that endogenous immune surveillance can be therapeutically reactivated when tumor-specific T cells are already present but functionally suppressed (3). However, the clinical benefit of ICB remains uneven across tumor types and individual patients, and primary, adaptive, and acquired resistance continue to limit its broader efficacy (4). These limitations indicate that the failure of immunotherapy is not simply due to insufficient antibody potency, but rather to defects across the cancer-immunity cycle, including inadequate antigen release, impaired antigen presentation, poor T-cell priming, defective lymphocyte trafficking, limited intratumoral infiltration, and terminal T-cell exhaustion (5).

A major biological explanation for ICB resistance is the presence of “immune-cold” or immune-excluded tumors, in which tumor-reactive lymphocytes are absent, spatially restricted to the stromal margin, or functionally disabled within the tumor microenvironment (TME) (6). In these settings, blocking PD-1/PD-L1 or CTLA-4 alone may be insufficient because the therapy depends on a pre-existing pool of antitumor T cells that can be reinvigorated. Tumors can also evade immunity by downregulating antigen-processing machinery, disrupting interferon signaling, recruiting regulatory T cells (Tregs) and myeloid-derived suppressor cells (MDSCs), polarizing tumor-associated macrophages (TAMs), and establishing hypoxic, acidic, and metabolically suppressive niches (7). In addition, stromal barriers, abnormal vasculature, dense extracellular matrix, and TGF-β-rich microenvironments can prevent cytotoxic T lymphocytes from reaching malignant cells even when systemic immune activation has occurred (7). Thus, effective immunotherapy increasingly requires not only immune checkpoint inhibition, but also local antigen generation, dendritic cell activation, T-cell recruitment, and reversal of suppressive TME programs.

Another unresolved issue is the systemic toxicity associated with broad immune activation. Immune checkpoint inhibitors can induce immune-related adverse events because checkpoint pathways also maintain peripheral tolerance and prevent excessive immune-mediated tissue damage (8). This concern becomes more pronounced when ICB is combined with cytokines, STING agonists, IDO inhibitors, TLR agonists, or other immunomodulators that may amplify immune activation beyond the tumor site (8). Therefore, an ideal next-generation immunotherapeutic strategy should activate immunity in a tumor-confined and temporally controlled manner, while minimizing off-target immune stimulation in normal tissues. This need has motivated the development of externally triggered therapeutic systems capable of converting physical energy into localized immune modulation, among which ultrasound-responsive nanoplatforms are mechanistically well suited to this purpose because they combine deep-tissue accessibility, noninvasiveness, spatial focusability, and on-demand activation (9).

Recent experimental studies support this shift from systemic immune modulation to ultrasound-engineered local immune activation. Semiconducting polymer nanobodies have been designed to remain relatively inactive before ultrasound exposure and to release anti-CTLA-4 antibodies *in situ* after singlet oxygen-mediated linker cleavage, thereby coupling sonodynamic tumor injury with localized checkpoint blockade. Sono-driven semiconducting polymeric nanoagonists have further demonstrated that ultrasound can trigger both immunogenic cell death (ICD) and STING agonist release, producing spatially restricted innate immune activation in head and neck squamous cell carcinoma (10). In orthotopic hepatocellular carcinoma, an activatable sulfur dioxide nanosonosensitizer enabled ultrasound-controlled sono-gaseous checkpoint trimodal therapy, illustrating how gas therapy, SDT, and immune modulation can be integrated within a single spatiotemporally gated platform. More recently, ultrasound-responsive cerasomal nanoparticles carrying the STING agonist SR-717 showed that stable nanocarriers can prevent premature immune agonist leakage and release their payload at the tumor site under ultrasound irradiation, enhancing dendritic cell maturation and CD8^+^ T-cell infiltration in triple-negative breast cancer. Collectively, these studies suggest that the central value of ultrasound-responsive nanoplatforms is not merely to intensify tumor cell killing, but to engineer the timing, location, and magnitude of immune activation within the TME.

Ultrasound has several intrinsic features, including noninvasiveness, deep-tissue penetration, spatial focusability, and adjustable acoustic parameters, that make it particularly well suited for cancer immunotherapy (11, 12). Unlike light-based modalities, whose tissue penetration is limited by optical scattering and absorption, ultrasound can reach deep-seated lesions and can be focused into defined anatomical regions with adjustable acoustic parameters (13, 14). Depending on frequency, intensity, duty cycle, and the presence of acoustic sensitizers or microbubbles, ultrasound can generate mechanical stress, acoustic cavitation, sonoporation, local heating, vascular permeabilization, and reactive oxygen species (ROS), all of which may influence tumor cell death and immune remodeling. These properties allow ultrasound to act simultaneously as an imaging-compatible physical trigger, a tumor-disruptive energy source, and a regulator of drug delivery within the TME (9, 15).

When integrated with nanotechnology, ultrasound can convert otherwise passive drug carriers into activatable immunotherapeutic systems. Nanoplatforms can improve the pharmacokinetics, tumor accumulation, and co-delivery of sonosensitizers, immune agonists, checkpoint inhibitors, cytokine-encoding nucleic acids, gas donors, or metabolic modulators, while ultrasound provides the external trigger for payload release or functional activation (9, 16). This design logic is particularly important for immunotherapy because many immune agonists are powerful but toxic when distributed systemically. By restricting their activation to the sonicated tumor region, ultrasound-responsive nanoplatforms may increase the therapeutic index of immunomodulatory agents and reduce unintended immune activation in normal tissues.

Mechanistically, ultrasound-responsive nanoplatforms can promote antitumor immunity through several interconnected routes. First, sonodynamic activation of nanosensitizers produces ROS that induce ICD, leading to the exposure or release of calreticulin, HMGB1, ATP, tumor antigens, and other danger signals that support dendritic cell maturation and T-cell priming (17). Second, ultrasound-mediated mechanical effects and cavitation can enhance vascular permeability and local delivery, thereby improving the intratumoral distribution of nanoparticles, antibodies, mRNA, and immune agonists. Third, ultrasound-triggered release can be engineered to occur only under defined acoustic stimulation, enabling temporal control over checkpoint blockade, STING activation, cytokine expression, gas therapy, or metabolic interference. Fourth, ultrasound-responsive systems can remodel physical and biochemical barriers in the TME, including hypoxia, dense extracellular matrix, IDO-mediated immunosuppression, and poor cytotoxic T-cell infiltration.

Several representative studies illustrate these principles. Catalytical nano-immunocomplexes achieved remote-controlled sono-metabolic checkpoint trimodal therapy, showing that ultrasound can coordinate catalytic tumor injury, metabolic reprogramming, and immune checkpoint modulation within a single platform (18). Activatable semiconducting polymer pro-nanomodulators were designed for orthotopic pancreatic cancer and used ultrasound to release a PD-L1 blocker and an IDO inhibitor, directly addressing the deep location and immunosuppressive nature of pancreatic tumors (19). Sono-activatable semiconducting polymer nanoreshapers further combined hyaluronidase-mediated matrix remodeling, H_2_S-based hypoxia relief, SDT-induced ICD, and IDO inhibition to improve immune infiltration in orthotopic pancreatic cancer. Ultrasound-responsive lipid nanosonosensitizers with size reduction and nitric oxide release provided another strategy for pancreatic tumors by enhancing tumor penetration, normalizing vascular and stromal barriers, inducing ICD, and improving the efficacy of anti-PD-L1 therapy. In parallel, ultrasound-responsive cerasomal nanoparticles demonstrated that robust carrier stability and ultrasound-triggered STING agonist release can be combined to drive type I interferon signaling and T-cell infiltration in triple-negative breast cancer.

The rapidly expanding literature has generated several recent reviews addressing ultrasound-responsive nanomedicine, including ultrasound-responsive drug delivery systems, physicochemical feature-directed nanocarrier design, ultrasound-responsive nanosystems for tumor immunotherapy, and stimuli-responsive strategies for sonodynamic immunotherapy (9, 20–23). Existing articles have mainly focused on ultrasound-triggered drug delivery and nanocarrier design, physicochemical response mechanisms, sonosensitizer-mediated sonodynamic therapy, or multimodal combinations of SDT with immunotherapy. These reviews have established an important foundation for the field, but the emerging ability of ultrasound-responsive systems to control the spatial location, temporal sequence, and biological stage of antitumor immune activation warrants a more explicitly immune-centered framework.

The present review therefore adopts a different perspective by conceptualizing ultrasound-responsive nanoplatforms as programmable systems for spatiotemporal immune engineering. Rather than asking only which payloads can be delivered or which forms of tumor cell death can be induced, we examine how ultrasound controls where, when, and in what sequence immune activation occurs across the cancer-immunity cycle (5). This perspective connects externally triggered nanoplatform activation with successive biological events ranging from local tumor injury and antigen release to innate immune sensing, antigen presentation, T-cell priming, intratumoral immune infiltration, and checkpoint regulation (24).

Accordingly, we organize the field around four interconnected dimensions: spatial confinement of immune activation to the insonated tumor; temporal gating of payload release and immune stimulation; sequential coordination of tumor-cell injury, innate immune sensing, antigen presentation, T-cell priming, and checkpoint relief; and tumor-specific matching between nanoplatform function and anatomical or immunological barriers. Within this framework, immunogenic cell death, pyroptosis, ferroptosis, necroptosis, cGAS–STING signaling, checkpoint blockade, cytokine mRNA delivery, gas therapy, and tumor-microenvironment remodeling are not treated as isolated therapeutic modules, but as components of a coordinated immune program. This distinction is particularly important because immunogenic tumor-cell death becomes therapeutically meaningful only when cell stress and death are successfully coupled to antigenicity, adjuvanticity, antigen presentation, and subsequent adaptive immune activation (25). Collectively, this framework shifts the emphasis from ultrasound-enhanced drug delivery toward the rational engineering of when, where, and how antitumor immunity is initiated, amplified, and sustained. **Figure 1** illustrates the central concept of this review: ultrasound-responsive nanoplatforms use focused acoustic energy to coordinate local tumor injury, immune activation, and therapeutic release within the tumor microenvironment. This strategy shifts cancer immunotherapy from systemic exposure toward spatially confined and temporally programmable immune engineering.programwhiletowardprogram.

**Figure 1:**
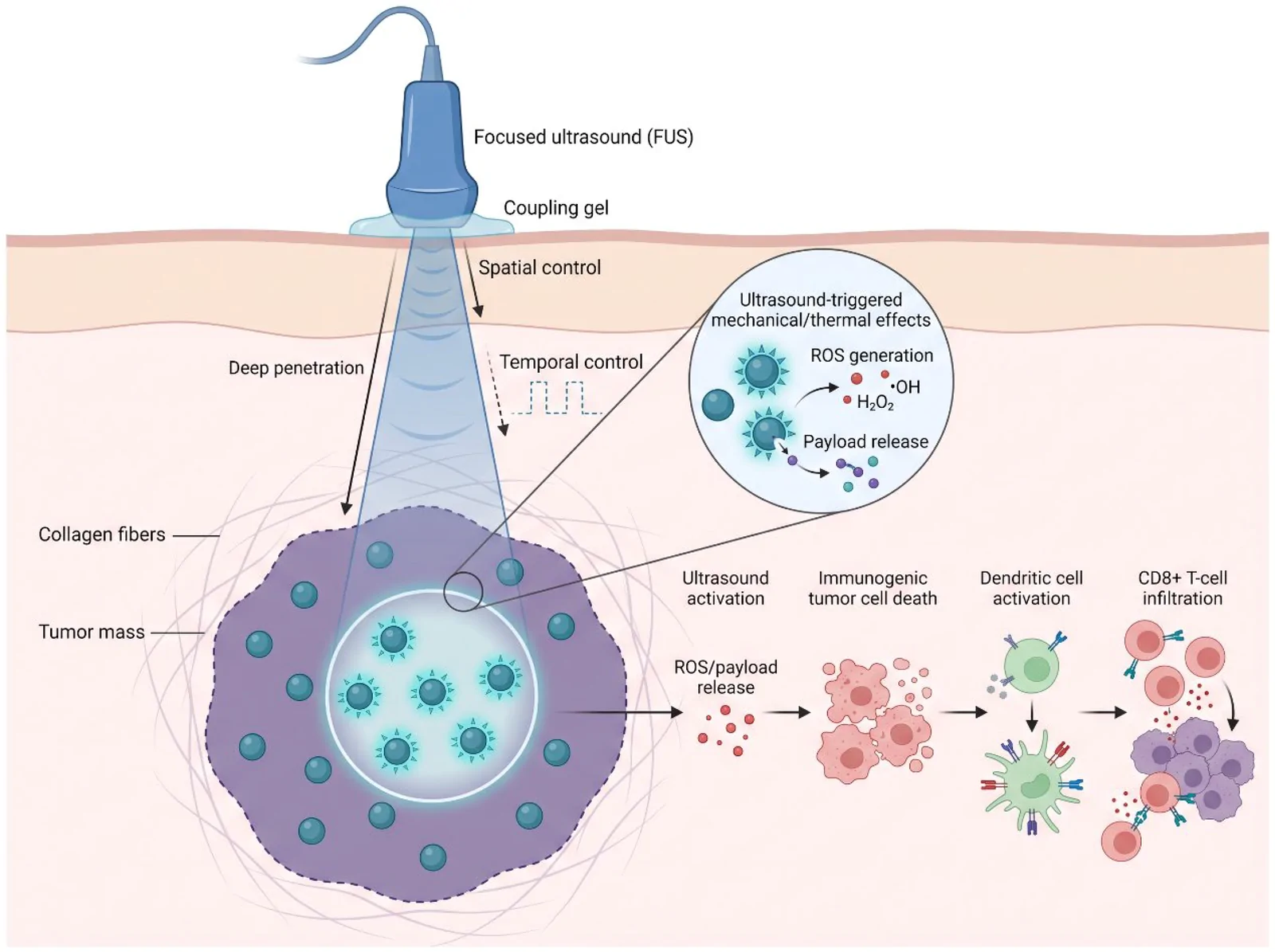
Conceptual overview of ultrasound-responsive cancer immunotherapy. Focused ultrasound activates nanoplatforms within deep tumors, triggering ROS generation, payload release, immunogenic cell death, dendritic cell activation, and CD8^+^ T-cell infiltration with spatial and temporal control.

## 2 Design principles of ultrasound-responsive immunotherapeutic nanoplatforms

### 2.1 Sonosensitizer-based nanoplatforms

Sonosensitizer-based nanoplatforms constitute the material foundation of ultrasound-responsive cancer immunotherapy (26–30). Upon ultrasound irradiation, these agents convert acoustic energy into cytotoxic reactive oxygen species (ROS), including singlet oxygen, hydroxyl radicals, and superoxide anions, through mechanisms involving acoustic cavitation, sonoluminescence, electron transfer, and local mechanical perturbation (9, 13, 16). Unlike conventional photodynamic therapy, sonodynamic therapy (SDT) can be activated in deep tissues with non-invasive and spatially focused ultrasound, allowing ROS production to be restricted to the tumor region and temporally synchronized with immune intervention.

Classical sonosensitizers such as chlorin e6 (Ce6), IR780, porphyrin derivatives, and porphyrin lipids remain widely used because of their established ROS-generating capacity and relative ease of nanocarrier integration (31, 32). For example, Ce6-loaded multifunctional nanodrugs have been used to induce SDT-mediated immunogenic cell death while simultaneously inhibiting TGF-β signaling to remodel the immunosuppressive tumor microenvironment. IR780-based nanosonodynamic systems have also been combined with anti-PD-L1 therapy to enhance immune activation in hepatocellular carcinoma models. These platforms illustrate a central principle of ultrasound-responsive immunotherapy: the sonosensitizer is not merely a cytotoxic module, but an upstream immune ignition unit.

New-generation sonosensitizers are increasingly designed with higher acoustic responsiveness, better tumor selectivity, and programmable immune functions. Semiconducting polymer nanoplatforms are particularly attractive because their π-conjugated backbones can be engineered for efficient ultrasound-triggered ROS generation, activatable drug release, and molecular imaging within a single architecture (10, 19, 33). In one representative system, a semiconducting polymer nanobody was engineered to remain relatively silent before ultrasound exposure and to release anti-CTLA-4 nanobodies after local ROS generation, thereby coupling SDT-induced tumor antigen release with checkpoint blockade in a spatially confined manner. A related semiconducting polymeric nanoagonist incorporated the STING agonist MSA-2 through a singlet-oxygen-cleavable linker, enabling ultrasound-triggered ROS production, immunogenic tumor damage, and synchronized STING activation in head and neck squamous cell carcinoma.

Inorganic and hybrid sonosensitizers provide another important design route. Metal-organic frameworks (MOFs) can integrate porphyrinic ligands, metal nodes, immune adjuvants, and biomimetic coatings, thereby functioning as both sonosensitizers and immunomodulatory depots (17, 34). A mitochondria-targeted, cancer-cell-membrane-biomimetic MOF system based on Zr-TCPP and R837 enhanced mitochondrial oxidative injury, promoted dendritic cell activation, and improved checkpoint blockade efficacy. ZnO-based nanoplatforms add an additional layer of immune modulation because ultrasound-triggered ROS generation and Zn²^+^ release can cooperate with chemotherapy-induced DNA damage to activate the cGAS-STING pathway (35). These examples show that inorganic components can be selected not only for acoustic energy conversion, but also for ion-mediated innate immune activation.

The immunological consequences of sonosensitizer activation extend beyond direct tumor killing. ROS generated by SDT can provoke endoplasmic reticulum stress, mitochondrial dysfunction, lysosomal disruption, and nuclear or mitochondrial DNA damage, all of which may contribute to immunogenic cell death and innate immune sensing (36–39). Tumor cells undergoing SDT-induced oxidative stress can expose calreticulin, release ATP and HMGB1, and provide tumor-associated antigens for dendritic cell uptake and cross-presentation (36, 37). In newer platforms, ROS can also serve as an intracellular trigger for pyroptosis, ferroptosis, or STING activation, thereby transforming ultrasound-induced oxidative injury into a broader immune-amplifying event (40, 41).

A key design trend is the transition from constitutively active nanomedicines to ultrasound-activated systems that remain functionally restrained before insonation and become active only after tumor-localized ultrasound exposure. This “silent-before-activation” principle is intended to reduce systemic immune toxicity while increasing the local concentration of immunogenic signals inside the tumor microenvironment (42). For example, sulfur dioxide nanosonosensitizers based on Aza-BODIPY chemistry were designed to undergo glutathione- and ultrasound-responsive activation, thereby combining sonodynamic therapy, gaseous therapy, and checkpoint blockade in an orthotopic hepatocellular carcinoma model. Similarly, ultrasound-responsive cerasome nanoparticles were used to oxidize lipid bilayers and trigger the local release of the STING agonist SR-717, improving STING-driven immunotherapy in triple-negative breast cancer.

### 2.2 Ultrasound-triggered release systems

Ultrasound-triggered release systems expand the role of ultrasound from an energy source for ROS generation to an external command signal for drug delivery, gene delivery, gas release, and immune checkpoint modulation (43–46). In these platforms, ultrasound can induce payload release through acoustic cavitation, microbubble destruction, membrane permeabilization, sonoporation, ROS-cleavable linkers, lipid oxidation, nanoparticle size transformation, and mechanical disruption of carrier structures (9, 13). This externally controlled release is especially valuable for immunotherapy because cytokines, checkpoint inhibitors, STING agonists, and inflammatory gases can produce systemic toxicity when distributed broadly, but may be highly effective when confined to the tumor site (2, 4).

Checkpoint blockade can be incorporated into ultrasound-responsive nanoplatforms to improve local immune activation while reducing off-target immune exposure (19, 33)while. The semiconducting polymer nanobody system releasing anti-CTLA-4 nanobodies after ultrasound irradiation exemplifies this strategy, as ultrasound-induced singlet oxygen both damaged tumor cells and cleaved the linker responsible for nanobody release (33). This design couples antigen release, danger signaling, and checkpoint inhibition in the same treated region, addressing the limitation that immune checkpoint blockade alone may fail when tumor antigenicity and T-cell priming are insufficient.

Nucleic acid delivery is another rapidly emerging application of ultrasound-triggered release. Ultrasound-targeted microbubble destruction can transiently increase vascular and cellular permeability, facilitating mRNA delivery into tumor tissues while providing contrast-enhanced ultrasound imaging guidance (47). A PD-L1-targeted IL-15 mRNA nanotherapy combined with ultrasound-targeted microbubble destruction enhanced local IL-15 expression, promoted immune effector activation, and integrated PD-L1 targeting with ultrasound-mediated delivery (47). This approach is conceptually important because it shifts ultrasound-responsive immunotherapy from passive drug release toward programmable local expression of immune cytokines.

STING agonists are particularly well suited for ultrasound-triggered release because systemic STING activation can cause dose-limiting inflammation, whereas local STING activation can promote type I interferon production, dendritic cell maturation, and CD8^+^ T-cell recruitment (39, 48, 49). Ultrasound-triggered release of MSA-2 from semiconducting polymer nanoagonists enabled synchronized SDT and STING pathway activation (10). ZnO-based nanoplatforms further demonstrated that ultrasound-enhanced ROS production, chemotherapy-induced DNA damage, and Zn²^+^ release can cooperate to amplify cGAS-STING signaling (35). More recently, cerasome nanoparticles were used to release SR-717 in response to ultrasound-induced lipid oxidation, strengthening antitumor immunity in triple-negative breast cancer (50).

Gas-releasing ultrasound-responsive systems add another layer of spatiotemporal immune regulation. Nitric oxide (NO) can modulate tumor vasculature, improve drug penetration, alter hypoxia, and cooperate with ROS-mediated tumor killing when released locally (51). Ultrasound-responsive lipid nanosonosensitizers with size reduction and NO release improved intratumoral penetration and supported synergistic sonodynamic-chemo-immunotherapy in pancreatic tumors (51). Sulfur dioxide (SO_2_)-releasing nanosonosensitizers similarly used tumor-associated glutathione and ultrasound activation to generate localized gaseous therapy while enhancing checkpoint-based immune responses (42). Together, these studies suggest that ultrasound-triggered release can be used to choreograph when and where cytotoxic, inflammatory, and immune-checkpoint-modulating signals appear in the tumor microenvironment. **Table 1** provides a comparative assessment of representative experimental studies rather than a simple catalog of platform composition. Importantly, therapeutic outcomes across these studies should not be directly compared without considering substantial heterogeneity in tumor models, host immune status, ultrasound parameters, treatment schedules, immune readouts, and safety assessments. We therefore compare not only the nanoplatform design and proposed immune mechanism, but also the experimental context, strength of functional immune evidence, safety evaluation, and major methodological limitations. This distinction is important for separating mechanistic proof-of-concept from more robust evidence of reproducible and durable antitumor immunity. **Figure 2** summarizes the modular engineering logic of ultrasound-responsive nanoplatforms. By integrating acoustic sensitizers with release-responsive immune payloads, these systems can convert ultrasound exposure into coordinated ROS generation, drug release, gas therapy, or local gene expression.

**Table 1:** Comparative evaluation of representative ultrasound-responsive nanoplatforms for cancer immunotherapy.

First author, year	Platform/immune strategy	Experimental model	Representative ultrasound parameters	Key immune evidence	Functional immune validation	Safety assessment	Major limitation/critical consideration	References
Zeng, 2022	Semiconducting polymer nanobody; SDT + ultrasound-gated anti-CTLA-4 release	Syngeneic 4T1 breast tumor model	1 MHz; 1.5 W/cm²; 50% duty cycle; 5 min	ICD; increased CTL activity; reduced intratumoral Tregs	Lung-metastasis inhibition, survival benefit, tumor rechallenge, and memory T-cell analysis	Hemocompatibility and short-term tolerability evaluated	Strong functional immune evidence, but primarily validated in a single murine tumor system; long-term nanomaterial clearance remains unclear	(33)
Luo, 2022	Cancer-cell-membrane-coated Zr-TCPP MOF + R837; mitochondrial SDT + CTLA-4 blockade	Bilateral 4T1 breast cancer model; additional orthotopic/metastatic evaluation	*In vivo*: 3 MHz; 1.5 W/cm²; 50% duty cycle	ICD, DC maturation, antigen presentation, enhanced checkpoint response	Bilateral tumor response and systemic immune activation	Mainly short-term preclinical tolerability	Biomimetic coating, MOF, adjuvant, mitochondrial targeting, and ICB increase manufacturing and mechanistic complexity	(17)
Zhang, 2022	Catalytical nano-immunocomplex containing sonosensitizer, anti-PD-L1 and adenosine deaminase	Bilateral 4T1 and CT26 syngeneic tumor models	1 MHz; 1.2 W/cm²; 50% duty cycle; 6 min	Reduced adenosine signaling; increased immune-effector activity; reduced immunosuppression	Primary/distant tumor control and metastasis evaluation	Body weight and histological evaluation performed	Strong combination design, but multiple active components make the individual contribution of metabolic reprogramming, SDT and ICB difficult to disentangle	(18)
Jiang, 2023	Semiconducting polymeric nanoagonist + MSA-2; ultrasound-gated STING activation	SCC7 head and neck squamous cell carcinoma model	50 kHz; 1 W/cm²; 50% duty cycle	ICD; STING–TBK1–IRF3 activation; IFN-I; DC maturation; effector T-cell infiltration	Systemic antitumor response and long-term immunological memory	Primarily preclinical short-term evaluation	Particularly strong mechanistic design, but the 50-kHz regimen differs substantially from MHz-range protocols used in many other studies, limiting direct cross-study comparison	(10)
Li, 2023	Semiconducting polymer pro-nanomodulator; ultrasound-triggered PD-L1 blocker + IDO inhibitor release	Orthotopic Panc02 pancreatic cancer in immunocompetent C57BL/6 mice	1 MHz; 1.2 W/cm²; 50% duty cycle; 5 min	ICD plus simultaneous reversal of PD-L1- and IDO-mediated immunosuppression	Orthotopic tumor eradication, survival analysis and metastasis suppression	Short-term *in vivo* tolerability evaluated	Clinically relevant orthotopic model is a strength; however, validation remains largely confined to one murine pancreatic model	(19)
Li, 2023	Sono-activatable nanoreshaper incorporating HAase, H_2_S donor and IDO inhibitor	Orthotopic pancreatic cancer model	1 MHz; 1 W/cm²; 50% duty cycle; 5 min	ECM degradation, hypoxia relief, ICD, IDO inhibition and increased immune-cell infiltration	Orthotopic tumor suppression and reduced metastasis	Mainly short-term tolerability	Multistage TME remodeling is biologically attractive but highly complex; the relative contribution of HA degradation, H_2_S, SDT and IDO inhibition is difficult to separate	(52)
Zhang, 2024	PMSN/DPMSN nanosonosensitizer; SDT-induced pyroptosis ± doxorubicin and anti-PD-L1	Orthotopic HepG2 liver tumor model for local efficacy; bilateral Hepa1–6 immunocompetent model for immune evaluation	500 kHz; 400 kPa; 25% duty cycle; approximately 2 min	GSDMD/GSDME-associated pyroptosis, inflammatory cell death and increased T-cell infiltration	Suppression of both insonated and distant tumors in bilateral immune-competent model	Body weight monitored; excessive acoustic exposure was explicitly considered during parameter optimization	Important model heterogeneity: orthotopic efficacy was assessed using HepG2 tumors in nude mice, whereas systemic immunity was evaluated using Hepa1–6 tumors in immunocompetent mice; these findings therefore cannot be mechanistically equated without caution	(40)
Wei, 2024	IR780@FOM-cRGD nanosonodynamic therapy + anti-PD-L1	Murine HCC model	1.0 W/cm²; 100% duty cycle; nine 20-s cycles separated by 30-s intervals	ICD-associated tumor injury and enhanced antitumor immune activation with PD-L1 blockade	Tumor-growth inhibition and immune profiling	Body weight and body temperature monitored	Demonstrates combination efficacy, but durable systemic immunity, rechallenge protection and long-term memory were not the principal endpoints	(32)
Liang, 2025	GSH-responsive Aza-DNBS SO_2_ nanosonosensitizer + anti-PD-L1	Orthotopic H22-Luc HCC and lung-metastasis model	*In vivo*: 1.5 MHz; 1 W/cm²; 50% duty cycle; 3 min	ICD, SO_2_-mediated redox disruption and immunosuppressive-TME reprogramming	Orthotopic tumor suppression and delayed lung metastasis	Blood and major organs collected for safety assessment; short-term evaluation	Orthotopic design strengthens relevance, but repeated gas generation plus checkpoint blockade requires more extensive long-term inflammatory and systemic safety evaluation	(42)
Fang, 2025	IR&ZnPc@LNP-NO; sequential size reduction/NO release followed by SDT + chemotherapy + anti-PD-L1	Orthotopic pancreatic cancer model	Low-dose: 1 MHz, 0.8 W/cm², 50%, 4 min; high-dose: 1 MHz, 1 W/cm², 100%, 5 min	ECM/vascular remodeling, ICD and increased CTL infiltration	Survival improvement in orthotopic pancreatic cancer	Preclinical tolerability assessed	Sequential low-/high-dose acoustic programming is innovative but increases procedural complexity and may complicate inter-device reproducibility and clinical standardization	(51)

**Figure 2:**
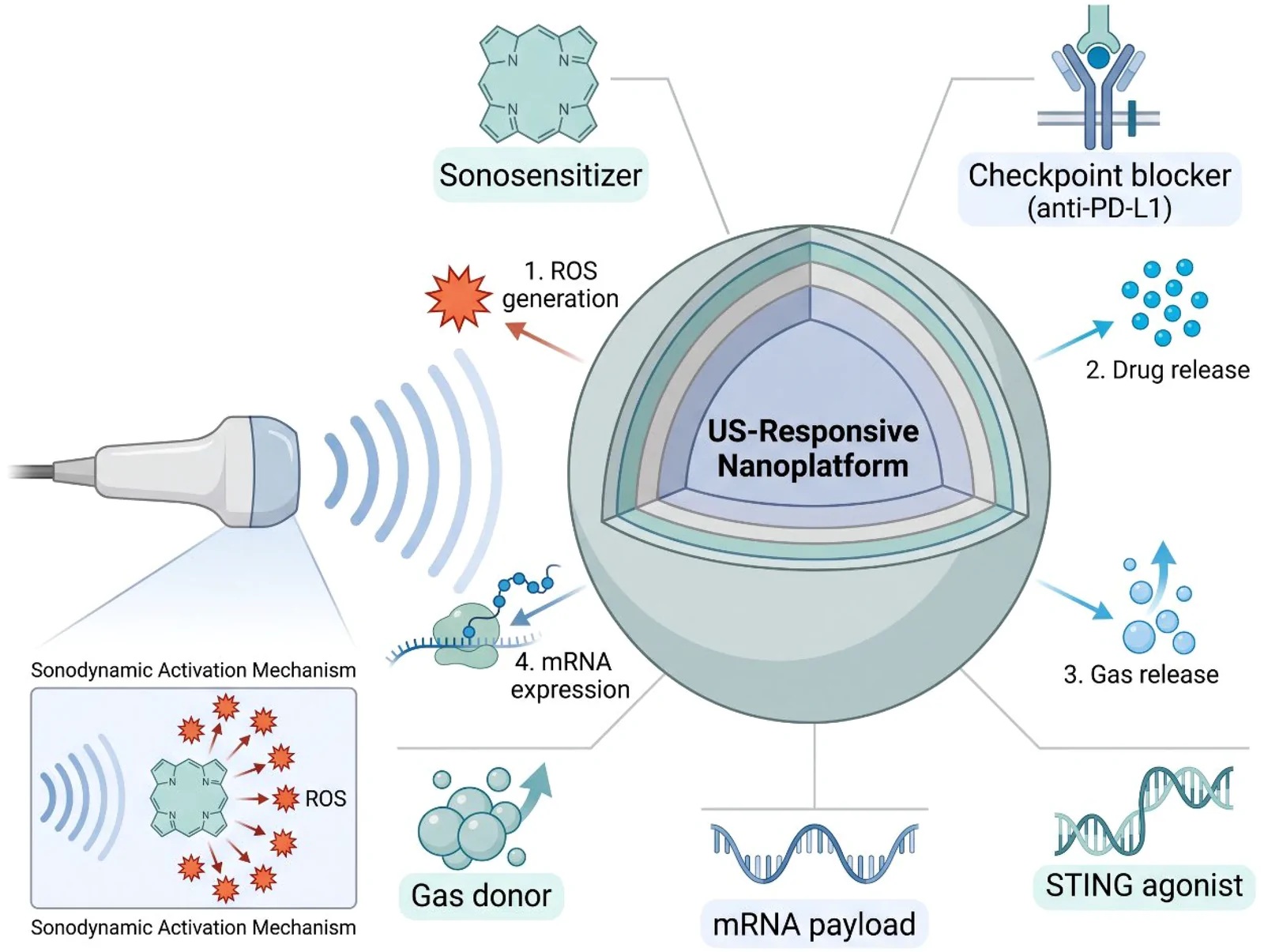
Modular design of ultrasound-responsive immunotherapeutic nanoplatforms. Representative platforms integrate sonosensitizers, checkpoint blockers, STING agonists, mRNA payloads, and gas donors to achieve ultrasound-triggered ROS generation, therapeutic release, and local immune modulation.

## 3 Ultrasound-induced immunogenic cell death as an *in situ* vaccine

### 3.1 ICD induced by sonodynamic therapy

Immunogenic cell death (ICD) is one of the most important biological bridges between ultrasound-induced tumor destruction and adaptive antitumor immunity (36, 37, 53). In contrast to non-immunogenic tumor cell death, ICD is characterized by the coordinated emission of damage-associated molecular patterns, including calreticulin exposure on the cell surface, ATP secretion, HMGB1 release, and type I interferon-associated inflammatory signaling (36, 37). These signals promote dendritic cell recruitment, antigen uptake, dendritic cell maturation, and cross-presentation of tumor antigens to cytotoxic T lymphocytes (5, 37). Therefore, in ultrasound-responsive nanoplatforms, tumor cell death should be viewed as the starting point of immune activation rather than the final therapeutic endpoint.

SDT is a powerful ICD-inducing modality because ultrasound-activated ROS can damage multiple intracellular compartments. ROS can induce endoplasmic reticulum stress, disrupt mitochondrial function, damage lysosomes, and promote nuclear or mitochondrial DNA leakage, thereby generating both antigenic substrates and danger signals for innate and adaptive immunity (35, 53–55). In the tumor microenvironment, this process can convert acoustically targeted tumor cells into an *in situ* source of antigens and adjuvant-like signals, initiating the cancer-immunity cycle. However, ICD alone is often insufficient to overcome established immune suppression, particularly in tumors with poor T-cell infiltration, high TGF-β activity, suppressive myeloid populations, or strong PD-1/PD-L1 signaling (4).

Several ultrasound-responsive nanoplatforms have therefore combined SDT-induced ICD with immune checkpoint blockade or microenvironmental remodeling. A mitochondria-targeted biomimetic MOF platform induced mitochondrial oxidative injury under ultrasound, promoted ICD, delivered the immune adjuvant R837, and enhanced anti-CTLA-4 therapy (17). A Ce6-containing multifunctional nanodrug combined SDT with TGF-β inhibition, thereby using ultrasound-induced ICD to supply antigenic stimulation while relieving a key immunosuppressive pathway in colorectal cancer and liver metastasis models (31). In hepatocellular carcinoma, IR780-based nanosonodynamic therapy combined with anti-PD-L1 treatment increased immune activation, supporting the idea that SDT-induced ICD can sensitize tumors to checkpoint blockade (32).

The coupling of ICD with STING activation further strengthens the vaccine-like effect of SDT (35, 56, 57). Chemotherapy and SDT can increase cytosolic DNA availability, while Zn²^+^ release from ZnO-based nanoplatforms can enhance cGAS-STING signaling, leading to type I interferon production and improved dendritic cell activation (35). An ultrasound-activated nanoplatform capable of inducing diverse forms of tumor cell death also remodeled the tumor microenvironment and improved immunotherapy efficacy, emphasizing that ICD can be amplified by combining oxidative stress with additional cell-death and immune-activation pathways (41). These studies collectively indicate that the most effective ultrasound-responsive immunotherapies do not rely on ROS cytotoxicity alone, but use SDT-induced ICD as the initiating event for a broader immune cascade.

### 3.2 Pyroptosis, ferroptosis, necroptosis and multi-modal cell death

Recent ultrasound-responsive nanoplatforms are moving beyond classical apoptosis-like oxidative tumor killing toward regulated cell-death programs with stronger inflammatory or immunogenic consequences (40, 41, 58, 59). Pyroptosis, ferroptosis, and necroptosis can generate distinct danger signals, inflammatory mediators, membrane-disruptive events, and antigen-release patterns, making them attractive mechanisms for converting local tumor destruction into systemic antitumor immunity (60–62). This shift is important because the immunological quality of tumor cell death can determine whether local therapy produces durable immune memory or merely transient cytoreduction.

Pyroptosis is characterized by gasdermin-mediated membrane pore formation, inflammatory cytokine release, cell swelling, and rapid liberation of intracellular contents (63, 64). Engineered extracellular vesicles delivering GSDMD-N mRNA were shown to induce pyroptosis and enhance antitumor immunotherapy, demonstrating that mRNA-based control of cell-death machinery can be used as an immune-amplifying strategy (65). In liver cancer, a nanosonosensitizer-augmented SDT system combined with PD-L1 blockade induced pyroptosis and strengthened antitumor immunity, suggesting that ultrasound-triggered oxidative stress can be paired with inflammatory cell-death programs to improve checkpoint responsiveness (40).

Necroptosis provides another inflammatory route for ultrasound-enhanced immunotherapy. This regulated necrotic process is commonly associated with RIPK1, RIPK3, and MLKL signaling and can release immunostimulatory danger signals that support dendritic cell activation and T-cell priming (66, 67). In glioblastoma, biomimetic calcium carbonate nanoparticles delivered IL-12 mRNA and used ultrasound to induce necroptosis, thereby combining local tumor injury with cytokine-driven immune activation in a brain tumor setting (59). This example is particularly relevant for deep-seated or anatomically protected tumors because ultrasound can provide external control while mRNA delivery supplies a programmable immune payload.

Ferroptosis is an iron-dependent form of regulated cell death driven by lipid peroxidation and redox imbalance (68, 69). In the context of cancer immunotherapy, ferroptotic tumor cells may modulate antigen release, dendritic cell function, T-cell activity, and the oxidative state of the tumor microenvironment, although its immune consequences may depend on timing, tumor type, and the balance between immune activation and immunosuppression (68, 70). The MIL-53@CM-272 platform illustrates the therapeutic potential of integrating ferroptosis with additional death and immune mechanisms, as it was designed to improve CM-272-primed bladder cancer immunotherapy through coordinated apoptosis, ferroptosis, and ICD induction (34).

Multi-modal cell-death induction may be especially valuable because tumors often resist single-pathway stress. An ultrasound-activated nanoplatform capable of inducing diverse cell-death modalities remodeled the tumor microenvironment and improved immunotherapy, supporting the concept that apoptosis, ICD, pyroptosis, ferroptosis, and necroptosis can be rationally combined to generate broader immune activation (41). Rather than asking whether SDT kills tumor cells efficiently, the next generation of ultrasound-responsive immunotherapeutic nanoplatforms should ask what type of death is induced, when it occurs, which innate sensors are engaged, and whether the resulting inflammatory context supports durable T-cell immunity.

However, the strength of evidence supporting these immune effects varies considerably among studies. Increased calreticulin exposure, ATP or HMGB1 release, inflammatory cytokine production, or intratumoral CD8^+^ T-cell accumulation provides useful mechanistic evidence of immunogenic stress, but these endpoints alone do not establish durable systemic antitumor immunity. More rigorous validation requires functional evidence, including immune-cell depletion experiments, control of untreated distant tumors, tumor rechallenge, long-term survival, and the generation of memory T-cell responses. Accordingly, the induction of ICD, pyroptosis, ferroptosis, or necroptosis should not automatically be interpreted as evidence of superior antitumor immunity, because the immunological outcome is strongly influenced by the tumor model, host immune context, timing and magnitude of cell death, and the endpoints used for evaluation.

## 4 Activation of innate immunity by ultrasound-responsive nanoplatforms

### 4.1 cGAS-STING pathway activation

The cGAS-STING pathway has become one of the most compelling innate immune targets for ultrasound-responsive cancer immunotherapy. Cytosolic double-stranded DNA derived from damaged nuclei, mitochondria, or tumor cells is detected by cyclic GMP-AMP synthase (cGAS), which produces cGAMP to activate STING on the endoplasmic reticulum (48). Activated STING recruits TBK1 and IRF3, leading to type I interferon production, inflammatory chemokine secretion, dendritic cell activation, and enhanced priming of tumor-specific CD8^+^ T cells (39, 48). In tumors, STING-dependent sensing of tumor-derived DNA is a key mechanism linking local cell damage to spontaneous antitumor T-cell immunity (71).

Ultrasound-responsive nanoplatforms are well positioned to activate this pathway because SDT can generate DNA damage and mitochondrial stress in a spatially confined manner. ROS generated under ultrasound can cause nuclear DNA damage, mitochondrial DNA leakage, and immunogenic stress signals, thereby increasing the cytosolic DNA substrates required for cGAS activation (35, 72). When this endogenous DNA sensing is combined with exogenous STING agonists or STING-amplifying ions, ultrasound becomes a tool for spatiotemporal innate immune activation rather than simply a cytotoxic trigger (10, 50).

Sono-driven STING activation has been demonstrated using semiconducting polymeric nanoagonists that integrate SDT and MSA-2 delivery. Under ultrasound, these nanoagonists produce singlet oxygen, induce tumor ICD, and release MSA-2 through ROS-responsive chemistry, thereby synchronizing antigen generation with STING agonism inside the treated tumor. This design is important because it addresses a major limitation of systemic STING agonist therapy: potent innate immune activation is desirable in the tumor microenvironment, but broad systemic activation may increase inflammatory toxicity (49, 73).

ZnO-based ultrasound-triggered platforms provide another strategy for STING amplification. In a PZnO@DOX system, ultrasound-enhanced ROS generation and doxorubicin-mediated DNA damage increased double-stranded DNA release, while Zn²^+^ release further promoted cGAS-STING pathway activation (35). This dual mechanism generated a stronger innate immune signal than chemotherapy or SDT alone and enhanced downstream dendritic cell maturation and T-cell activation. Cerasome nanoparticles loaded with SR-717 further illustrate the logic of ultrasound-gated STING agonist delivery, as ultrasound-induced oxidation of the lipid bilayer enabled controlled agonist release and improved STING-driven immunotherapy in triple-negative breast cancer (50).

Overall, the cGAS-STING axis provides a mechanistic framework for understanding how ultrasound-responsive nanoplatforms engineer local innate immunity. SDT supplies damage-associated DNA and oxidative stress; nanocarriers supply STING agonists, Zn²^+^, or other immune amplifiers; and focused ultrasound determines where and when these events occur. This spatiotemporal control may be particularly valuable for converting immunologically cold tumors into inflamed tumors with type I interferon signaling, dendritic cell maturation, and CD8^+^ T-cell infiltration (6, 71). Despite these encouraging findings, direct comparison among STING-oriented platforms remains difficult because the studies differ substantially in STING agonist chemistry, tumor model, ultrasound exposure, combination therapy, and immune endpoints. Moreover, increases in type I interferon signaling, dendritic cell maturation, or CD8^+^ T-cell infiltration do not necessarily demonstrate that STING activation is causally responsible for long-term tumor control. Future studies would benefit from STING-pathway loss-of-function experiments, immune-cell depletion, distant-tumor models, and rechallenge studies to distinguish pathway activation from functionally durable systemic immunity. **Figure 3** shows how ultrasound-induced tumor-cell death becomes the starting point of antitumor immunity. Instead of representing cell death as a terminal cytotoxic event, this schematic emphasizes its role in antigen release, innate immune sensing, and adaptive T-cell priming.

**Figure 3:**
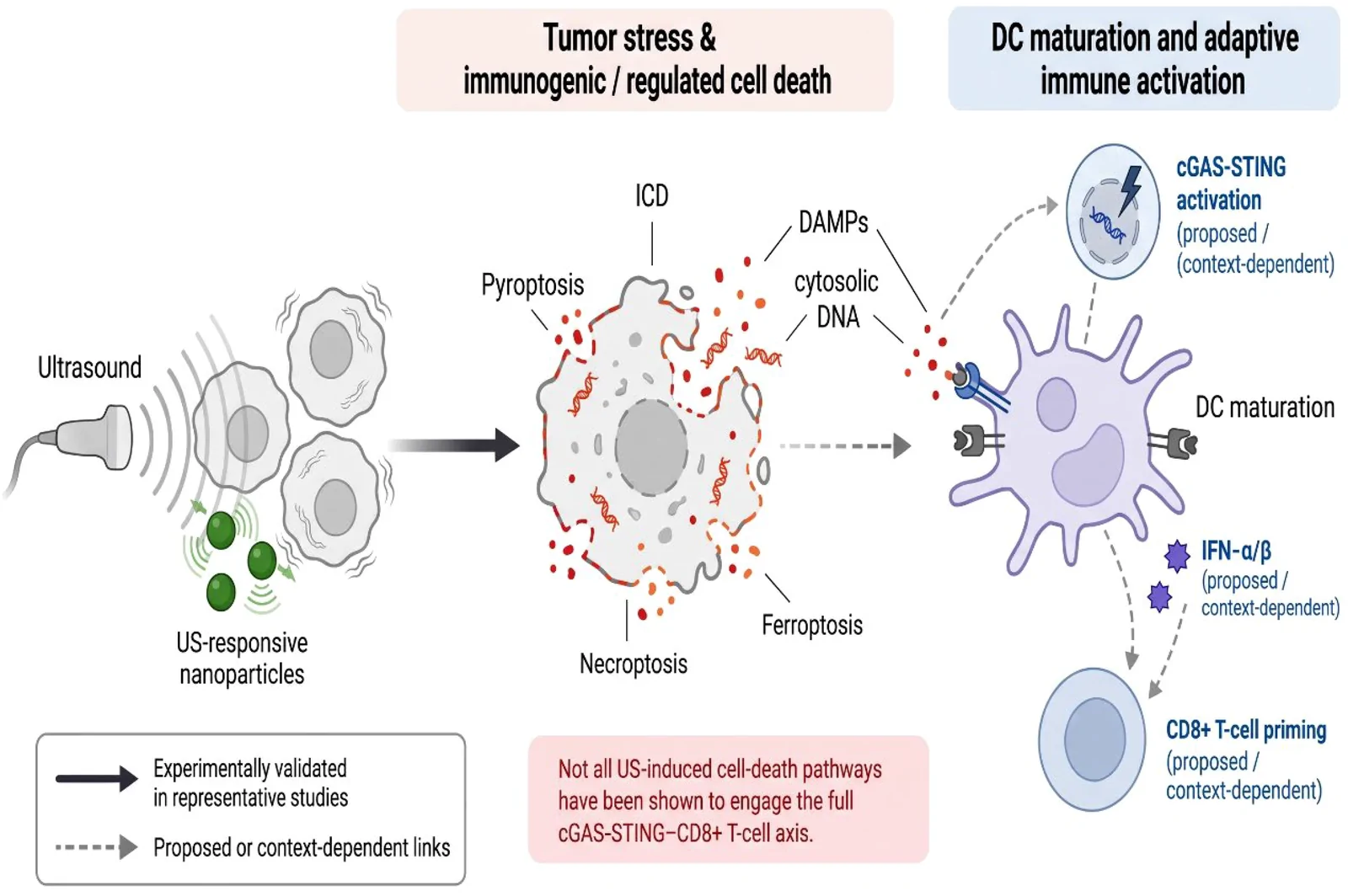
Ultrasound-induced immunogenic cell death and innate immune activation. Ultrasound-activated nanoplatforms induce ROS stress and regulated tumor-cell death, leading to DAMP release, cytosolic DNA sensing, cGAS-STING activation, dendritic cell maturation, and CD8^+^ T-cell priming.

### 4.2 Ultrasound-controlled cancer vaccination and immune priming

Ultrasound-controlled cancer vaccination is based on the concept that a treated tumor can be converted into an *in situ* vaccine factory. In this model, ultrasound-responsive nanoplatforms generate tumor antigens through local cell death, provide danger signals through ICD or inflammatory cell-death pathways, and deliver immune adjuvants, cytokines, or checkpoint modulators to support dendritic cell priming (5, 37). The goal is not only to eradicate insonated lesions, but also to initiate systemic immunity capable of controlling distant or recurrent disease.

For *in situ* vaccination to succeed, several events must be coordinated: tumor antigens must be released in an immunogenic context, dendritic cells must capture and present these antigens, lymph-node priming must generate tumor-specific T cells, and effector cells must infiltrate the tumor microenvironment. Ultrasound-responsive nanoplatforms are attractive because they can temporally align antigen release with adjuvant exposure and immune checkpoint modulation. This alignment is difficult to achieve with freely administered drugs, whose biodistribution and pharmacokinetics may not match the timing of tumor antigen generation (5, 17).

A mitochondria-targeted biomimetic MOF platform provides a representative example of ultrasound-controlled *in situ* vaccination (17). By combining cancer-cell-membrane coating, mitochondrial SDT, and R837-mediated immune adjuvanticity, this system promoted tumor antigen release, dendritic cell maturation, and checkpoint blockade-mediated T-cell responses (17). Because the tumor membrane coating supported homologous tumor targeting and the porphyrinic MOF enabled ultrasound-triggered ROS generation, the platform integrated antigen source, danger signal, and adjuvant delivery within a single localized therapeutic process.

mRNA delivery further broadens the vaccination concept by allowing ultrasound-responsive systems to induce local expression of immunostimulatory proteins. In glioblastoma, biomimetic calcium carbonate nanoparticles delivered IL-12 mRNA and used ultrasound-induced necroptosis to strengthen tumor immunogenicity, combining local inflammatory tumor death with cytokine-mediated immune activation (59). In another strategy, PD-L1-targeted IL-15 mRNA nanotherapy combined with ultrasound-targeted microbubble destruction promoted local IL-15 expression and improved tumor immunotherapy, showing that ultrasound can enhance both delivery efficiency and immune priming (47). These studies suggest that ultrasound-controlled vaccination can be engineered through either antigenic tumor destruction, cytokine expression, or both.

Therefore, ultrasound-responsive cancer vaccination should be understood as a programmable immune-priming strategy. The tumor supplies patient-specific antigens; the nanoplatform supplies sonosensitizers, adjuvants, cytokines, or checkpoint modulators; and ultrasound supplies spatial and temporal control. This triad directly matches the central premise of the review: ultrasound-responsive nanoplatforms are not simply enhanced drug carriers, but systems for engineering when, where, and how immune activation unfolds inside the tumor microenvironment (17, 47, 59). Because individual studies assess antitumor immunity using substantially different endpoints, increases in isolated immune markers should not be interpreted as equivalent evidence of therapeutic immunity. **Table 2** therefore compares the major ultrasound-responsive immune mechanisms according to their activation trigger, representative experimental models, immune readouts, level of immune evidence, functional validation, and principal limitations. We distinguish molecular evidence of immunogenic stress from cellular immune remodeling and more stringent functional evidence of systemic antitumor immunity, thereby avoiding overinterpretation of surrogate immune endpoints.

**Table 2:** Comparative strength of immune evidence across major ultrasound-responsive immunotherapeutic mechanisms.

Immune mechanism	Ultrasound-responsive trigger	Representative platform/payload	Representative tumor model	Primary immune-related readouts	Level of immune evidence*	Functional validation	Principal limitation/critical consideration	References
Immunogenic cell death (ICD)	Sonosensitizer-mediated ROS generation and organelle stress	Zr-TCPP(TPP)/R837@M; Ce6/galunisertib nanodrug	Syngeneic 4T1 breast cancer; colorectal cancer with liver metastasis	CRT/HMGB1-associated ICD, DC maturation, antigen presentation, increased effector T-cell responses	Molecular + cellular + functional systemic	Metastasis suppression, tumor rechallenge and memory T-cell responses were demonstrated in the biomimetic MOF study	ICD markers alone do not establish systemic immunity; combination with adjuvants or ICB makes the contribution of ICD itself difficult to isolate	(17)
cGAS–STING activation	Ultrasound-induced ROS/DNA stress combined with controlled STING-agonist release	Semiconducting polymeric nanoagonist–MSA-2; polymeric STING pro-agonist	HNSCC and other murine solid-tumor models	STING–TBK1–IRF3 activation, type I IFN production, DC activation, effector T-cell infiltration	Molecular + cellular + functional systemic	Systemic tumor control and long-term immunological memory reported in representative studies	Most evidence derives from engineered agonist-delivery systems; the extent to which endogenous ultrasound-induced DNA damage alone drives STING-dependent immunity remains context-dependent	(10, 56)
Pyroptosis	ROS-driven gasdermin activation during amplified SDT	PMSN/DPMSN nanosonosensitizer + anti-PD-L1	Orthotopic HepG2 liver tumor for local pyroptosis; bilateral Hepa1–6 model for immune evaluation	GSDMD/GSDME-associated pyroptosis, increased TILs, CD8^+^ T cells, DCs and inflammatory tumor-cell death	Molecular + cellular + functional distant-tumor evidence	Suppression of both insonated and untreated distant tumors; enhanced anti-PD-L1 efficacy	Local pyroptosis and systemic immunity were evaluated in different tumor/host models; durable memory and causal immune-depletion evidence remain limited	(40)
Necroptosis	Ultrasound-associated CaCO_3_ nanoparticle activation and necroptotic tumor-cell death	IL-12 mRNA@cRGD-CM-CaCO_3_ nanoparticles	Orthotopic GL261 glioblastoma model	ATP/HMGB1 release, DC maturation, IL-12/IFN-γ expression and CD8^+^ T-cell infiltration	Molecular + cellular + functional local	Improved tumor control and survival; durable cures reported in a subset of treated mice	No tumor-rechallenge or immune-cell-depletion study was used to establish long-term systemic immune dependence	(59)
Ferroptosis-associated immune activation	Fe³^+^-mediated redox disruption, GSH depletion and enhanced ROS generation	MIL-53@CM-272 + anti-PD-1	Murine bladder cancer model	Ferroptosis/apoptosis, ICD-associated stress, DC maturation and anti-PD-1 sensitization	Molecular + cellular	Enhanced tumor control in combination with checkpoint blockade	Ferroptosis, apoptosis and ICD occur simultaneously, making the immune contribution of ferroptosis difficult to isolate; systemic memory was not established	(34)
Localized checkpoint blockade	Ultrasound-generated ¹O_2_ cleaves responsive linker and releases checkpoint inhibitor locally	Semiconducting polymer nanobody carrying anti-CTLA-4	Murine solid-tumor model using 4T1 cells in mechanistic studies	Increased CTL activity, reduced Treg-mediated suppression and SDT-associated ICD	Cellular + functional systemic	Tumor regression, metastasis inhibition, rechallenge protection and durable immune memory	Strong functional evidence, but checkpoint release and SDT-induced ICD occur concurrently, complicating attribution of benefit to local ICB alone	(33)
Metabolic checkpoint reprogramming	Ultrasound-gated release/activation of metabolic modulators together with SDT	Sono-metabolic nano-immunocomplex containing ADA + anti-PD-L1; PD-L1/IDO pro-nanomodulator	Bilateral 4T1/CT26 models; orthotopic pancreatic cancer	Reduced adenosine signaling or IDO-mediated suppression, increased effector T cells, reduced suppressive myeloid populations	Molecular + cellular + functional systemic	Bilateral tumor control, metastasis suppression and systemic effector-T-cell activation demonstrated in representative studies	Multi-component systems simultaneously modify metabolism, checkpoints and ICD; factorial designs are needed to define the contribution of each module	(18, 19)
Cytokine mRNA delivery	UTMD-mediated sonoporation or ultrasound-enhanced nanoparticle transfection	PD-L1-targeted IL-15 mRNA nanotherapy; IL-12 mRNA CaCO_3_ nanoparticles	Solid-tumor model for IL-15; orthotopic GL261 glioblastoma for IL-12	Local cytokine expression, immune-effector activation, ICD/necroptosis-associated priming and increased CD8^+^ T-cell infiltration	Cellular + functional antitumor; systemic evidence varies by platform	IL-15/UTMD study reported systemic immune activation and immune-memory-associated effects; IL-12 platform improved survival	Expression level alone does not establish durable immune programming; cell-type-specific transfection, expression duration, systemic cytokine exposure and repeated-dose safety require further study	(47, 59)
Gas-mediated immune remodeling	Ultrasound-triggered or TME-gated release of SO_2_, NO or H_2_S combined with SDT	Aza-DNBS SO_2_ nanosonosensitizer; IR&ZnPc@LNP-NO; H_2_S-containing nanoreshaper	Orthotopic HCC and pancreatic cancer models	ICD, redox disruption, hypoxia relief, vascular/ECM remodeling and increased CTL infiltration	Molecular + cellular + functional antitumor	Orthotopic tumor suppression and reduced metastatic progression reported in representative systems	Gas kinetics, local concentration and systemic exposure are difficult to standardize; immune effects are intertwined with vascular, metabolic and cytotoxic effects	(42, 51, 52)
Stromal-barrier remodeling	Ultrasound-gated HA degradation, nanoparticle size reduction and vascular modulation	HAase/H_2_S nanoreshaper; size-reducible NO-releasing lipid nanosonosensitizer	Orthotopic pancreatic cancer	ECM degradation, improved penetration/perfusion, hypoxia relief and increased CTL infiltration	Cellular + functional local	Improved penetration, tumor control and metastasis restriction in orthotopic models	Increased immune-cell infiltration may partly result from physical transport changes rather than direct immune reprogramming; causal separation remains limited	(51, 52)

## 5 Remodeling the immunosuppressive tumor microenvironment

### 5.1 Reversing hypoxia and stromal barriers

A major obstacle for ultrasound-responsive cancer immunotherapy is that many solid tumors, especially pancreatic ductal adenocarcinoma and other deep-seated desmoplastic tumors, are protected by dense stromal barriers, abnormal vasculature, high interstitial fluid pressure, and profound hypoxia (74, 75). In pancreatic cancer, excessive extracellular matrix deposition, hyaluronic acid accumulation, activated fibroblasts, and poor vascular perfusion limit nanoparticle penetration and restrict cytotoxic T-cell infiltration, thereby creating both a physical barrier and an immunological barrier to therapy (6). Hypoxia further reinforces immune suppression by promoting angiogenic imbalance, metabolic stress, myeloid-cell recruitment, and T-cell dysfunction, making stromal and oxygenation remodeling important strategies for improving immunotherapeutic outcomes (76–78).

Ultrasound-responsive nanoplatforms offer a distinctive opportunity to remodel these barriers because focused ultrasound can activate therapeutic functions only after the particles have reached the tumor region. Activatable semiconducting polymer pro-nanomodulators were designed to release a PD-L1 blocker and an IDO inhibitor under deep-tissue ultrasound irradiation, thereby linking local SDT with checkpoint and metabolic immune modulation in orthotopic pancreatic cancer (19). This design is important because it addresses two problems simultaneously: poor immune activation caused by insufficient tumor injury and persistent immune suppression caused by PD-L1 and IDO signaling (19, 79). By concentrating immune modulation at the insonated tumor site, this strategy also reduces the conceptual risk of systemic immune toxicity associated with broadly distributed checkpoint or metabolic inhibitors.

Tumor penetration can be further improved by ultrasound-triggered extracellular matrix remodeling. Sono-activatable semiconducting polymer nanoreshapers were engineered to multiply remodel the pancreatic tumor microenvironment through hyaluronidase-mediated degradation of hyaluronic acid and hydrogen sulfide-mediated hypoxia relief (52). Hyaluronic acid degradation can loosen the dense stromal meshwork and reduce diffusion barriers, while local H_2_S generation can alleviate hypoxia and enhance the susceptibility of tumor cells to oxidative stress-based therapy (52). In this context, ultrasound is not simply a trigger for tumor killing, but a spatial command that reshapes the stromal architecture and oxygenation state required for immune-cell entry.

A related approach uses ultrasound-responsive lipid nanosonosensitizers to improve intratumoral transport through size reduction and nitric oxide release (51). IR&ZnPc@LNP-NO was designed to undergo ultrasound-triggered size shrinkage and NO release, thereby improving penetration into pancreatic tumors and promoting cytotoxic T lymphocyte infiltration (51). NO can modulate vascular tone, improve perfusion, and cooperate with ROS-mediated tumor damage, which makes it particularly useful in poorly perfused and hypoxic pancreatic tumors (51). The combination of size adaptability, gas release, SDT, chemotherapy, and immune activation illustrates how ultrasound-responsive systems can convert hostile stromal niches into more permeable and immune-accessible tumor microenvironments.

Together, these studies suggest that reversing hypoxia and stromal barriers should be treated as a central design principle rather than an auxiliary function of ultrasound-responsive immunotherapy. For deep solid tumors, therapeutic success depends not only on whether a nanoplatform can generate ROS or release immunotherapeutics, but also on whether it can enter the tumor, spread through the matrix, restore local perfusion, and create routes for T-cell infiltration (19, 51, 52).

### 5.2 Targeting immunosuppressive cytokines and metabolic checkpoints

Beyond physical barriers, the tumor microenvironment is shaped by soluble cytokines and metabolic checkpoints that actively suppress antitumor immunity. TGF-β promotes immune exclusion, fibroblast activation, epithelial-mesenchymal transition, regulatory T-cell function, and resistance to PD-1/PD-L1 blockade (80, 81). IDO-mediated tryptophan catabolism depletes local tryptophan and accumulates kynurenine, thereby suppressing effector T-cell activity, promoting regulatory T-cell differentiation, and supporting tumor immune escape (79). Hypoxia, glutathione-rich redox buffering, lactate accumulation, and altered amino acid metabolism further reduce the effectiveness of ROS-based therapy and immune checkpoint blockade (75).

Ultrasound-responsive nanoplatforms can target these suppressive pathways by combining local tumor damage with timed release of immune modulators. Catalytical nano-immunocomplexes were developed for remote-controlled sono-metabolic checkpoint trimodal cancer therapy, integrating ultrasound-triggered activation with metabolic checkpoint modulation and immune enhancement (18). This strategy reflects an important conceptual shift: ultrasound-responsive therapy is no longer limited to inducing oxidative injury, but can also regulate tumor metabolism and immune escape pathways in a remotely controlled manner (18).

TGF-β blockade is a particularly relevant partner for SDT-induced immune activation. A multifunctional nanodrug combining Ce6-mediated SDT with TGF-β inhibition enhanced immune responses against colorectal cancer and liver metastasis, indicating that ultrasound-induced ICD can be strengthened when a major immunosuppressive cytokine axis is simultaneously suppressed (31). Mechanistically, SDT provides tumor antigens and danger signals, while TGF-β inhibition may reduce immune exclusion and permit more effective dendritic cell activation and T-cell infiltration. This combination is especially rational for tumors in which TGF-β-rich stroma physically and functionally excludes cytotoxic lymphocytes from tumor nests.

Metabolic immune checkpoints can also be targeted through ultrasound-triggered release. Activatable semiconducting polymer pro-nanomodulators released both a PD-L1 blocker and an IDO inhibitor after ultrasound exposure, thereby combining checkpoint blockade with tryptophan-metabolism reprogramming in orthotopic pancreatic cancer (19). Because IDO activity can suppress effector T cells and promote immune tolerance, local IDO inhibition may help sustain the T-cell responses initiated by SDT-induced antigen release. This type of platform is particularly valuable because it couples the timing of metabolic checkpoint inhibition to the timing of ultrasound-induced immune priming.

Redox and hypoxia modulation further broaden the immunometabolic scope of these systems. Sono-activatable polymer nanoreshapers used H_2_S generation to relieve hypoxia while remodeling hyaluronic acid-rich stroma, thereby improving the immune contexture of orthotopic pancreatic tumors (52). GSH-responsive sulfur dioxide nanosonosensitizers exploited the reducing tumor microenvironment to activate sono-gaseous therapy and checkpoint-based immune enhancement. These approaches suggest that tumor-associated hypoxia and redox imbalance can be transformed from barriers into biochemical triggers for site-specific immune activation. **Figure 4** depicts how ultrasound-responsive platforms remodel the hostile tumor microenvironment rather than simply killing tumor cells. This is especially relevant for pancreatic cancer and other desmoplastic tumors, where dense ECM, hypoxia, abnormal perfusion, and metabolic checkpoints limit immune infiltration. However, improved nanoparticle penetration or increased CD8^+^ T-cell infiltration should not necessarily be attributed solely to immune reprogramming, because ultrasound-induced changes in vascular permeability, cavitation, and stromal disruption may independently alter tumor access. Studies that directly separate these physical delivery effects from immune-mediated mechanisms remain limited.

**Figure 4:**
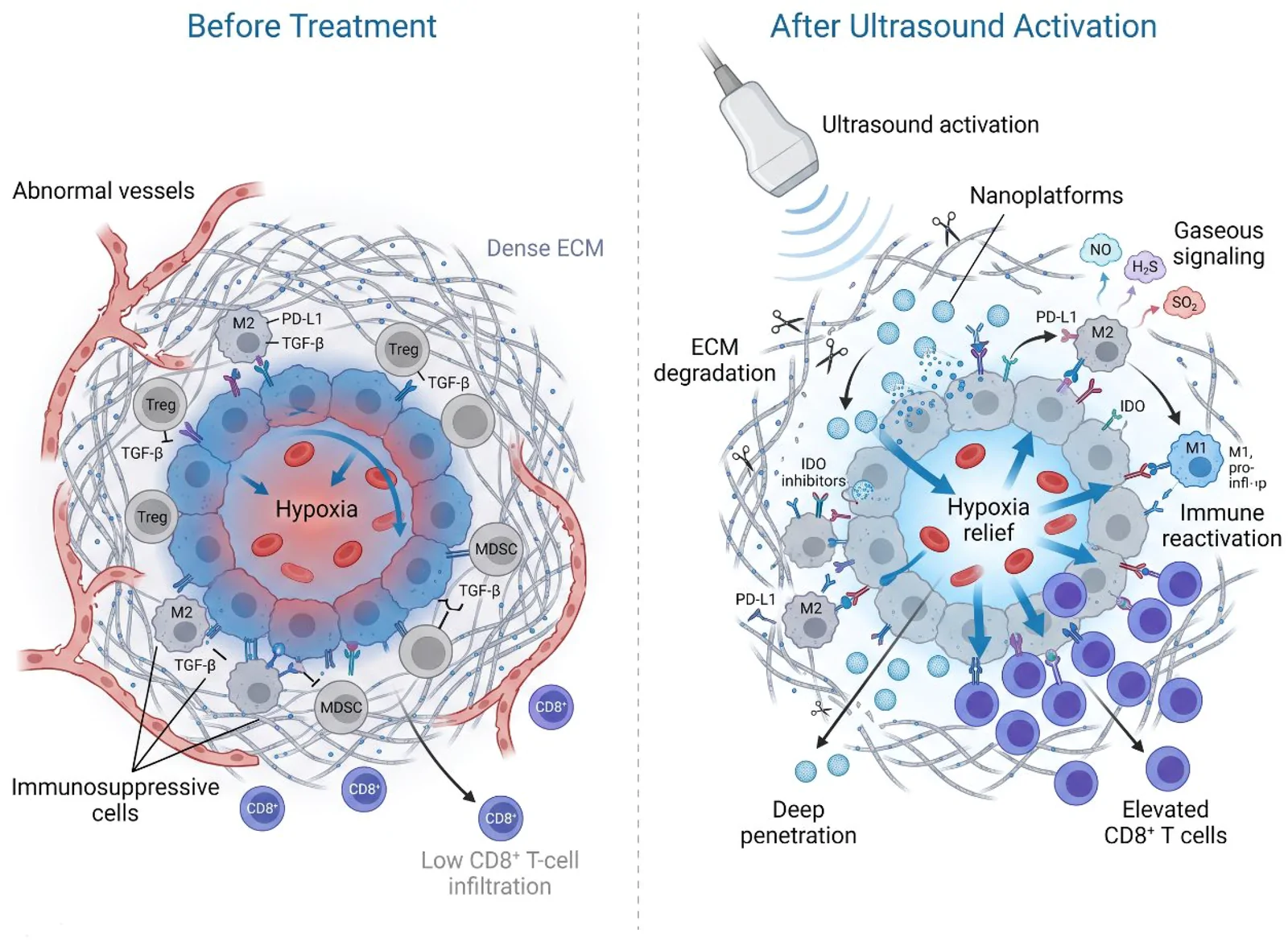
Tumor microenvironment remodeling by ultrasound-responsive nanoplatforms. Ultrasound-triggered release of matrix-remodeling enzymes, gas mediators, checkpoint blockers, and metabolic inhibitors can reduce stromal barriers, relieve hypoxia, suppress immune checkpoints, and enhance cytotoxic T-cell infiltration.

## 6 Ultrasound-responsive platforms combined with immune checkpoint blockade

### 6.1 Localized anti-CTLA-4 and anti-PD-L1 activation

Immune checkpoint blockade has transformed cancer therapy, but its efficacy remains limited by insufficient antigen release, poor T-cell infiltration, immune-excluded tumor architecture, and systemic immune-related adverse events (2, 4, 6). Ultrasound-responsive nanoplatforms provide a rational way to improve checkpoint therapy because they can first generate local tumor antigens and danger signals through SDT, then release or amplify checkpoint blockade within the same tumor region (32, 33, 42). This spatially coordinated strategy is particularly attractive because checkpoint antibodies are most effective when pre-existing or therapy-induced antitumor immunity is present.

Localized anti-CTLA-4 activation has been demonstrated using semiconducting polymer nanobodies. In this system, ultrasound irradiation triggered ROS generation and cleaved singlet-oxygen-responsive linkers to release anti-CTLA-4 nanobodies locally, thereby integrating SDT-induced ICD with checkpoint blockade (33). Because CTLA-4 blockade can enhance T-cell priming but may also cause systemic immune toxicity, ultrasound-gated local release provides a strategy to concentrate immune activation at the tumor site while limiting unnecessary exposure in normal tissues. This platform therefore embodies the review’s central concept of engineering spatial and temporal control over immune activation.

Anti-PD-L1 strategies have also been combined with ultrasound-responsive immunochemotherapy. An *in situ* micro-nano conversion platform augmented tumor-localized immunochemotherapy by improving drug retention and immune activation under ultrasound control (82). In hepatocellular carcinoma, nanosonodynamic therapy combined with anti-PD-L1 treatment enhanced tumor immune activation, supporting the idea that SDT-induced antigen release and PD-L1 blockade can cooperate to reinvigorate antitumor T-cell responses. These studies indicate that ultrasound can function as an upstream sensitizer for PD-1/PD-L1 blockade by increasing tumor immunogenicity before checkpoint inhibition is applied (82).

Gas-based ultrasound-responsive checkpoint combinations further expand this logic. An activatable sulfur dioxide nanosonosensitizer integrated SDT, SO_2_ gas therapy, and checkpoint modulation to achieve precisely controllable sono-gaseous checkpoint trimodal therapy in orthotopic hepatocellular carcinoma (42). SO_2_ generation and ROS production can intensify local tumor stress, while checkpoint blockade prevents the newly generated immune response from being suppressed by inhibitory ligand signaling. This trimodal design illustrates how ultrasound-responsive platforms can synchronize cytotoxic stress, inflammatory modulation, and checkpoint therapy within a defined treatment window.

### 6.2 Multi-checkpoint and immunosuppressive pathway blockade

Although single checkpoint blockade can be effective in selected tumors, many solid tumors use multiple suppressive pathways simultaneously. PD-L1 limits effector T-cell function, CTLA-4 suppresses T-cell priming, IDO remodels tryptophan metabolism, CD47 inhibits macrophage-mediated phagocytosis, and hypoxia or stromal TGF-β signaling reduces immune-cell infiltration (79, 80). Therefore, ultrasound-responsive platforms are increasingly designed to target more than one checkpoint or suppressive pathway in a coordinated manner (19, 51, 52).

The combination of PD-L1 blockade and IDO inhibition is a representative multi-pathway strategy. Activatable semiconducting polymer pro-nanomodulators released a PD-L1 blocker and an IDO inhibitor after ultrasound activation, thereby targeting both adaptive immune resistance and metabolic immune suppression in pancreatic cancer (19). This is mechanistically attractive because SDT can release tumor antigens and DAMPs, PD-L1 blockade can preserve effector T-cell function, and IDO inhibition can prevent tryptophan-depletion-driven T-cell suppression. The platform therefore links antigen generation, checkpoint release, and metabolic remodeling into one ultrasound-controlled therapeutic sequence (19, 79).

Stromal and hypoxia remodeling can be considered checkpoint-adjacent because stromal barriers restrict immune-cell infiltration, whereas hypoxia and metabolic stress impair immune-cell function within tumors (76, 80, 83). Sono-activatable nanoreshapers degraded hyaluronic acid and relieved hypoxia, thereby improving the tumor microenvironmental conditions required for immune-cell infiltration and checkpoint responsiveness. This strategy is especially relevant for pancreatic cancer, where dense ECM and hypoxia can prevent cytotoxic lymphocytes from reaching tumor cells even when checkpoint pathways are pharmacologically blocked (74, 75). By removing these upstream barriers, ultrasound-responsive matrix remodeling may increase the effective depth of checkpoint immunotherapy.

Ultrasound-triggered NO release also provides a multi-functional route to enhance checkpoint-based therapy. IR&ZnPc@LNP-NO combined size reduction, NO release, SDT, chemotherapy, and immunotherapy to improve pancreatic tumor penetration and increase cytotoxic T-cell infiltration. Because NO can influence vascular perfusion and tissue penetration, its ultrasound-controlled release may help overcome the transport barriers that limit both nanomedicine accumulation and immune-cell access. In this sense, multi-checkpoint and pathway blockade should be broadly defined to include not only PD-L1, CTLA-4, IDO, and CD47, but also stromal, vascular, metabolic, and redox pathways that determine whether checkpoint-reinvigorated immune cells can actually function within the tumor (51). Although combining SDT with checkpoint blockade consistently improves tumor control in many preclinical studies, the relative contribution of ultrasound-induced antigen generation versus checkpoint inhibition is often difficult to distinguish. Factorial experimental designs comparing ultrasound alone, nanoplatform alone, checkpoint blockade alone, and their combinations are therefore essential for establishing true therapeutic synergy rather than additive effects.

## 7 Ultrasound-mediated delivery of immunotherapeutic biomolecules

### 7.1 mRNA delivery

mRNA therapeutics provide a flexible way to express cytokines, immune activators, or cell-death executors directly within tumors, but their clinical use is limited by instability, poor tissue selectivity, endosomal trapping, and systemic inflammatory toxicity (47, 59, 65). Ultrasound-responsive delivery systems address these limitations by adding external spatial and temporal control to mRNA delivery, allowing gene expression to be enhanced primarily at the insonated tumor site (47, 84). This feature is especially important for potent immunostimulatory molecules such as IL-12 and IL-15, whose systemic exposure can produce substantial toxicity despite their strong antitumor activity (59).

IL-12 mRNA delivery has been used for glioblastoma sono-immunotherapy. Biomimetic calcium carbonate nanoparticles carrying IL-12 mRNA were engineered with tumor-targeting and ultrasound-responsive properties, enabling local immune activation in glioblastoma models. Ultrasound-induced necroptosis provided inflammatory tumor-cell death, while IL-12 mRNA expression enhanced antitumor immune activation, thereby combining controlled tumor injury with cytokine-driven immune stimulation. This approach is particularly meaningful for brain tumors because anatomical barriers and systemic toxicity make localized immune programming highly desirable (59).

mRNA can also be used to directly encode cell-death machinery. Engineered extracellular vesicles delivering GSDMD-N mRNA induced pyroptosis and enhanced cancer immunotherapy, demonstrating that mRNA delivery can convert tumor cells into inflammatory death factories (65). Although this system is not simply a conventional drug-delivery platform, it supports the broader principle that mRNA can be used to install programmable immunogenic death pathways in tumors (63, 65). When combined with ultrasound-responsive delivery or activation strategies, this concept could allow pyroptosis to be spatially confined to tumor tissues while reducing off-target inflammatory injury (65).

IL-15 mRNA delivery further demonstrates the value of ultrasound-mediated local transfection. A PD-L1-targeted IL-15 mRNA nanotherapy combined with ultrasound-targeted microbubble destruction enhanced local mRNA delivery, activated immune effector cells, and improved antitumor immunotherapy (47). This platform is especially elegant because PD-L1 targeting enriches the nanotherapy in immune-suppressive tumor regions, while ultrasound-targeted microbubble destruction increases local delivery efficiency and enables imaging-guided treatment. Therefore, ultrasound-mediated mRNA therapy should be viewed not merely as nucleic acid delivery, but as a way to spatially program cytokine expression and inflammatory immune activation in the tumor microenvironment (47).

### 7.2 Ultrasound-targeted microbubble destruction and sonoporation

Ultrasound-targeted microbubble destruction (UTMD) and sonoporation provide a physical strategy for enhancing local delivery of genes, mRNA, proteins, and nanotherapeutics (85–88). Microbubbles oscillate or collapse under ultrasound exposure, generating mechanical forces that transiently increase vascular permeability, disrupt cell membranes, and enhance endocytosis or cytosolic entry of therapeutic cargoes (84, 89, 90). Because these effects occur primarily within the ultrasound focal region, UTMD can improve local transfection while limiting systemic biodistribution of immunostimulatory biomolecules (84, 90).

In cancer immunotherapy, UTMD is especially useful because many immune biomolecules have narrow therapeutic windows. PD-L1-targeted IL-15 mRNA nanotherapy used UTMD to enhance local transfection and immune effector activation, showing how ultrasound can connect molecular targeting, image guidance, and immune cytokine delivery in one therapeutic workflow (47). In this model, ultrasound does not merely assist passive nanoparticle accumulation; it actively increases mRNA delivery efficiency and determines the anatomical site of cytokine expression (47). This local control is critical for cytokines such as IL-15, which can strongly stimulate immune effector cells but may cause toxicity if expressed systemically.

Sonoporation also creates a conceptual bridge between nanomedicine and cell therapy. Intraparticle double-scattering-decoded sonogenetics was developed to augment immune checkpoint blockade and CAR-T therapy, demonstrating that ultrasound-responsive genetic control can be integrated with advanced immunotherapeutic modalities (91). By enabling externally controlled activation of engineered immune pathways, sonogenetic platforms may help overcome poor tumor penetration, limited intratumoral persistence, and insufficient local activation of adoptively transferred immune cells (91). This direction expands ultrasound-responsive immunotherapy from drug release and ROS generation toward programmable control of cellular immune functions.

The broader implication is that ultrasound-mediated delivery can unify several therapeutic layers: mechanical permeabilization, image-guided localization, nanocarrier activation, nucleic acid transfection, and immune-cell programming (84, 89, 91). For future platforms, the key design question is not only whether ultrasound improves delivery efficiency, but whether it can align biomolecule release, gene expression, tumor-cell death, antigen presentation, and immune-cell activation within the same spatial and temporal window (91). This makes UTMD and sonoporation a crucial bridge between ultrasound-responsive nanomedicine and next-generation cancer immunotherapies such as mRNA therapy, cytokine therapy, checkpoint blockade, and CAR-T cell therapy (91). A further limitation is that increased intratumoral mRNA expression does not necessarily indicate efficient or durable immune programming. Transfection efficiency, cellular identity of the transfected population, duration of protein expression, systemic cytokine exposure, and repeated-dose tolerability should be evaluated when comparing ultrasound-assisted mRNA delivery strategies.

## 8 Cancer type-specific applications

### 8.1 Hepatocellular carcinoma

Hepatocellular carcinoma (HCC) provides a clinically relevant setting for ultrasound-responsive immunotherapy because it develops within a tolerogenic hepatic immune environment shaped by chronic inflammation, exhausted T cells, suppressive myeloid cells, regulatory T cells, stromal remodeling, and metabolic dysfunction (92, 93). The liver is also anatomically suitable for ultrasound-guided treatment, making HCC an attractive tumor type for testing sono-immunotherapeutic strategies in orthotopic models rather than only in superficial subcutaneous tumors. Because HCC often exhibits heterogeneous immune infiltration and incomplete responses to immune checkpoint blockade, local approaches that increase antigen release, induce inflammatory cell death, and enhance PD-1/PD-L1 blockade are particularly rational (93).

Recent HCC-focused studies highlight three major directions: SDT plus PD-L1 blockade, pyroptosis induction, and gas-enhanced sono-immunotherapy. A nanosonosensitizer-augmented SDT strategy combined with PD-L1 blockade induced pyroptosis and improved therapeutic efficacy against liver cancer, suggesting that inflammatory tumor-cell death can enhance checkpoint responsiveness (40). In another HCC model, nanosonodynamic therapy combined with anti-PD-L1 treatment increased immune activation, supporting the concept that SDT-induced immunogenic damage can sensitize liver tumors to checkpoint blockade (32). More recently, an activatable sulfur dioxide nanosonosensitizer integrated SDT, SO_2_ gas therapy, and checkpoint modulation to achieve precisely controllable sono-gaseous checkpoint trimodal therapy in orthotopic HCC (42).

These studies are important because they move beyond simple tumor ablation and instead use ultrasound to orchestrate local immune activation in an immunosuppressive organ context. Orthotopic HCC models are particularly valuable because the hepatic microenvironment preserves liver-specific vascular and immunological features and can generate tumor–host interactions and treatment responses that differ substantially from those observed in subcutaneous models (94, 95). For this reason, HCC represents a strong disease model for evaluating whether ultrasound-responsive platforms can convert local tumor destruction into systemic antitumor immunity while avoiding excessive liver inflammation (40, 42).

### 8.2 Pancreatic cancer

Pancreatic cancer is one of the clearest examples of a tumor type in which ultrasound-responsive nanoplatforms can demonstrate unique advantages (19, 51, 52). Pancreatic ductal adenocarcinoma is characterized by deep anatomical location, dense desmoplastic stroma, abundant extracellular matrix, hypovascularity, hypoxia, high interstitial pressure, and limited immune-cell infiltration (74, 75). These features restrict nanoparticle penetration, impair drug distribution, and generate an immune-cold or immune-excluded phenotype that limits the efficacy of conventional immunotherapy. Therefore, pancreatic cancer is a particularly suitable setting for platforms that combine deep tissue activation, stromal remodeling, hypoxia relief, and localized immune modulation (19, 51, 52).

Activatable semiconducting polymer pro-nanomodulators showed how ultrasound can be used to coordinate SDT with local release of a PD-L1 blocker and an IDO inhibitor in orthotopic pancreatic cancer (19). This design directly addresses the two dominant barriers in pancreatic tumors: insufficient local immune activation and strong metabolic/checkpoint-mediated immune suppression. Sono-activatable semiconducting polymer nanoreshapers further expanded this strategy by degrading hyaluronic acid through HAase activity and relieving hypoxia through H_2_S generation, thereby remodeling the physical and metabolic barriers that prevent immune-cell infiltration (52). Ultrasound-responsive lipid nanosonosensitizers with size reduction and NO release improved pancreatic tumor penetration and cytotoxic T lymphocyte infiltration, highlighting the value of ultrasound-triggered structural adaptation in dense tumors (51).

Together, these pancreatic cancer studies illustrate the central advantage of ultrasound-responsive immunotherapy: deep-tissue non-invasive activation can be coupled to microenvironment-adaptive drug release. In preclinical orthotopic models, ultrasound enables activation at tissue depths that are difficult to access using optical triggers. However, these findings do not by themselves establish clinical feasibility, because human pancreatic tumors differ substantially in depth, acoustic window, stromal composition, body habitus, and surrounding anatomy. Pancreatic cancer therefore represents an informative model for testing whether ultrasound-responsive systems can overcome deep-tissue and stromal barriers, rather than evidence that these platforms are already close to clinical translation (19, 51, 52).

### 8.3 Glioblastoma

Glioblastoma (GBM) poses a distinct set of delivery and immune challenges because the blood-brain barrier and blood-tumor barrier restrict therapeutic access to intracranial tumors (96, 97). Although the blood-tumor barrier can be partially disrupted in GBM, its permeability is spatially heterogeneous, leaving invasive tumor regions and immune-suppressive niches difficult to reach (96, 98). In addition, the central nervous system immune environment, steroid exposure, myeloid dominance, and profound T-cell dysfunction collectively limit the efficacy of conventional immunotherapy in GBM (98, 99).

Ultrasound-responsive nanoplatforms provide a mechanistically interesting strategy for GBM because focused ultrasound may facilitate localized delivery while limiting systemic exposure (100–102)while. Nevertheless, intracranial acoustic transmission, blood–brain barrier heterogeneity, neurotoxicity, and local inflammatory responses introduce additional translational constraints that are not adequately captured by extracranial tumor models. Biomimetic calcium carbonate nanoparticles carrying IL-12 mRNA were developed for targeted GBM sono-immunotherapy, combining tumor-homing delivery, ultrasound-induced necroptosis, and local cytokine expression (59). In this platform, ultrasound-triggered necroptotic tumor-cell death provides inflammatory danger signals, while IL-12 mRNA expression supports antitumor immune activation inside the tumor microenvironment (59). This design is conceptually strong because it uses ultrasound not only as a delivery enhancer, but also as a local immune-programming signal in a tumor type where systemic immunotherapy has been difficult to translate (98).

For future GBM applications, ultrasound-responsive nanoplatforms will need to solve both delivery and safety challenges. The ideal platform should cross or transiently bypass the BBB/BTB, distribute into infiltrative tumor margins, activate only within insonated regions, and avoid excessive neuroinflammation (96, 98). Therefore, GBM may benefit most from biomimetic carriers, mRNA payloads, focused ultrasound-mediated barrier modulation, and tightly controlled acoustic dosing (96, 103).

### 8.4 Breast cancer and TNBC

Breast cancer, especially triple-negative breast cancer (TNBC), is another important application area for ultrasound-responsive immunotherapy (50, 104, 105). TNBC has relatively higher immunogenicity than hormone receptor-positive breast cancer, but clinical responses to immune checkpoint blockade remain limited and biomarker-dependent (106–108). Because TNBC lacks hormone receptors and HER2 amplification, new strategies are needed to increase local immune activation, improve T-cell infiltration, and enhance responsiveness to checkpoint therapy (107, 109).

Earlier breast tumor studies using mitochondria-targeted cancer-cell-membrane-biomimetic MOF-mediated SDT demonstrated that ultrasound-triggered mitochondrial oxidative stress can induce immunogenic tumor damage and cooperate with checkpoint blockade (17). This type of biomimetic platform is relevant to breast cancer because membrane coating can improve homologous tumor targeting, while mitochondrial SDT can intensify ROS-induced ICD and immune priming. More recently, ultrasound-responsive cerasome nanoparticles loaded with the STING agonist SR-717 improved STING-driven immunotherapy in TNBC (50). This platform is especially timely because STING activation can promote type I interferon signaling, dendritic cell maturation, and CD8^+^ T-cell infiltration, which are all desirable for converting poorly inflamed TNBC lesions into more immune-responsive tumors (50, 71).

The TNBC setting also highlights the importance of local control. Systemic STING activation can produce off-target inflammatory toxicity, whereas insufficient intratumoral innate immune activation may limit checkpoint responsiveness (110, 111). Ultrasound-responsive STING agonist release offers a possible solution by confining innate immune activation to the insonated tumor region and synchronizing STING signaling with ultrasound-induced tumor damage (50). Therefore, TNBC represents a relevant disease context for evaluating ultrasound-gated innate immune agonists and checkpoint-sensitizing nanoplatforms (50).

### 8.5 Colorectal cancer, bladder cancer and other solid tumors

Beyond HCC, pancreatic cancer, GBM, and breast cancer, ultrasound-responsive immunotherapeutic nanoplatforms have also been applied to colorectal cancer, bladder cancer, and other solid tumors. These tumor types are useful for exploring how SDT-induced ICD, TME remodeling, and multi-modal cell death can be adapted to different immune landscapes (31, 34, 41). In colorectal cancer and liver metastasis models, a multifunctional nanodrug combined SDT with TGF-β inhibition, thereby using ultrasound-induced ICD to initiate immune activation while suppressing a major stromal immunosuppressive cytokine pathway. This is particularly relevant because TGF-β signaling can drive immune exclusion and resistance to checkpoint blockade in solid tumors (80, 81).

Bladder cancer provides another context in which multi-modal immune remodeling may improve therapeutic responses. The MIL-53@CM-272 platform was designed to improve CM-272-primed bladder cancer immunotherapy through coordinated induction of apoptosis, ferroptosis, and ICD (34). This strategy supports the broader idea that ultrasound-responsive or nanomedicine-based immunotherapy should not rely only on one form of tumor-cell death, but instead can integrate multiple regulated death pathways to produce stronger immune stimulation (34, 70). An ultrasound-activated nanoplatform that induced diverse cell-death modalities further demonstrated that remodeling the tumor microenvironment through combined cell-death mechanisms can improve immunotherapy efficacy across solid tumor models (41).

These cancer type-specific studies collectively show that ultrasound-responsive immunotherapy is not a single disease-specific technique, but a modular therapeutic logic. In immune-cold tumors, it can increase antigen release and innate immune activation through mechanisms such as ICD and STING signaling (10, 56). In stromal tumors, particularly pancreatic cancer, ultrasound-responsive platforms can improve intratumoral penetration, degrade extracellular matrix barriers, relieve hypoxia, and remodel the tumor microenvironment (51, 52). In checkpoint-resistant tumors, ultrasound can synchronize ICD with spatially controlled immune checkpoint blockade or metabolic checkpoint inhibition (19, 33). In anatomically protected tumors such as glioblastoma, focused ultrasound can facilitate localized drug delivery and transient BBB/BTB modulation (100). The next step is therefore to match platform design to tumor-specific biological and anatomical barriers rather than applying the same sonosensitizer–carrier architecture across all cancer types. Because different tumor types present distinct anatomical, stromal, vascular, and immunological barriers, ultrasound-responsive nanoplatforms should be matched to cancer-specific therapeutic needs. Because therapeutic efficacy in a preclinical tumor model does not by itself establish clinical translatability, **Table 3** evaluates representative tumor-specific platforms using a more cautious and objective framework. In addition to the biological barrier and therapeutic rationale, we consider clinically relevant model validation, material composition and potential clearance, manufacturing complexity, compatibility with controllable ultrasound exposure, pharmacological and safety requirements, and the major evidence gaps that remain before clinical evaluation. These considerations are intended to identify translational requirements rather than to rank individual platforms as more or less clinically promising.

**Table 3:** Tumor-specific applications and objective translational considerations of representative ultrasound-responsive immunotherapeutic nanoplatforms.

Cancer type	Major biological/anatomical barrier	Representative ultrasound-responsive strategy	Preclinical validation/therapeutic rationale	Objective translational considerations	Key evidence gaps before clinical translation	References
Hepatocellular carcinoma	Immunosuppressive liver TME; limited checkpoint response	IR780@FOM-cRGD nanosonodynamic therapy + anti-PD-L1	SDT-induced ICD combined with checkpoint blockade enhanced antitumor immune activation in a murine HCC model	Ultrasound accessibility of liver lesions and combination with an established checkpoint modality provide a clinically recognizable treatment concept	Requires validation in additional orthotopic/immunocompetent models; pharmacokinetics, repeated-dose toxicity, long-term clearance, and clinically standardized acoustic exposure remain to be established	(32)
Hepatocellular carcinoma	Weak inflammatory immune activation and checkpoint resistance	Pyroptosis-inducing PMSN nanosonosensitizer + anti-PD-L1	Ultrasound-enhanced oxidative stress induced pyroptosis and improved local and distant tumor control with PD-L1 blockade	Sub-10-nm silicate-based design incorporated renal-clearance considerations; bilateral tumor experiments provided evidence beyond local tumor inhibition	Durability of immune memory, repeated-dose safety, human-scale acoustic feasibility, and reproducible clearance require further evaluation	(40)
Hepatocellular carcinoma	Hypoxia, redox buffering, and immunosuppression	Activatable Aza-BODIPY SO_2_ nanosonosensitizer + checkpoint therapy	Integrated SDT, GSH-responsive SO_2_ release, and checkpoint modulation in orthotopic HCC	Orthotopic validation and ultrasound-gated gas generation improve biological relevance and local controllability	Gas-release kinetics, systemic exposure, pharmacokinetics, repeated-dose inflammatory toxicity, material clearance, and scalable manufacturing require further assessment	(42)
Pancreatic cancer	Deep anatomical location; strong PD-L1/IDO-mediated immunosuppression	Activatable semiconducting polymer pro-nanomodulator releasing PD-L1 blocker + IDO inhibitor	Ultrasound-triggered ROS generation synchronized ICD with local release of two immunomodulators in orthotopic pancreatic cancer; deep-tissue activation was demonstrated experimentally	Orthotopic validation and externally gated drug release directly address deep-tissue treatment and systemic immune-exposure concerns	Human pancreatic acoustic windows, quantitative PK/clearance, batch reproducibility, long-term safety, and scalability of the multifunctional polymer system remain unresolved	(19)
Pancreatic cancer	HA-rich dense ECM, hypoxia, poor immune-cell penetration, IDO activity	HAase/H_2_S/IDO-inhibitor sono-activatable polymer nanoreshaper	HA degradation, hypoxia relief, SDT-induced ICD, and IDO inhibition were integrated in an orthotopic pancreatic model	The platform addresses multiple barriers that are characteristic of pancreatic cancer rather than relying solely on increased cytotoxicity	Multi-component architecture increases manufacturing and quality-control complexity; relative contributions of individual modules, PK, biodegradation, and long-term safety require clarification	(52)
Pancreatic cancer	Poor penetration, dense ECM, abnormal vasculature and limited CTL infiltration	Size-reducible IR&ZnPc@LNP-NO with sequential NO release, SDT, chemotherapy and immunotherapy	Low-dose ultrasound promoted size reduction and NO-mediated TME remodeling; higher-dose ultrasound induced ROS and ICD in orthotopic pancreatic tumors	Lipid-based formulation and externally programmable sequential activation provide clinically recognizable material and energy-delivery concepts	Two-stage acoustic dosing increases procedural complexity; reproducibility across ultrasound devices, long-term NO-related safety, PK, clearance, and manufacturing scale-up require evaluation	(51)
Glioblastoma	BBB/BTB, intracranial localization, immune suppression and systemic cytokine toxicity	Cancer-cell-membrane-coated CaCO_3_ nanoparticles delivering IL-12 mRNA	Ultrasound-induced necroptosis was combined with local IL-12 mRNA expression and brain-tumor targeting	Biodegradable CaCO_3_ and local mRNA expression provide potentially favorable design features for reducing systemic cytokine exposure	Intracranial acoustic transmission, neurotoxicity, biodistribution, duration of cytokine expression, repeated-dose tolerability and validation in more clinically representative GBM models require further study	(59)
Triple-negative breast cancer	Insufficient innate immune activation and incomplete response to ICB	SR-717-loaded ultrasound-responsive cerasome	Ultrasound-induced membrane oxidation triggered local STING-agonist release, enhancing type I IFN signaling, DC maturation and CD8^+^ T-cell infiltration	Ultrasound-gated agonist release may reduce uncontrolled systemic exposure compared with constitutively available innate immune agonists	Systemic inflammatory toxicity, repeated-dose STING activation, PK, clearance of the cerasomal carrier, manufacturing reproducibility and long-term safety remain to be established	(50)
Breast cancer	Insufficient tumor targeting, ICD and immune priming	Cancer-cell-membrane-coated mitochondria-targeted Zr-TCPP MOF + R837 + anti-CTLA-4	Mitochondrial SDT induced ICD, R837 promoted DC maturation, and checkpoint blockade enhanced systemic antitumor effects and memory responses	Bilateral and metastatic models provide relatively strong functional immune validation	MOF composition, mitochondrial targeting, membrane coating, adjuvant loading and checkpoint therapy create substantial manufacturing complexity; biodegradation, clearance and long-term safety need further characterization	(17)
Bladder cancer	Hypoxia, redox protection and insufficient immune priming	MIL-53@CM-272 integrating SDT, ferroptosis, apoptosis and anti-PD-1 sensitization	Fe³^+^-based MOF improved ROS generation and oxygenation while CM-272 contributed epigenetic modulation and ICD	TME-responsive degradation and integration of multiple immune-relevant cell-death pathways provide mechanistic rationale	Complexity of MOF degradation products, PK/clearance, chronic metal exposure, manufacturing reproducibility and whether multimodal killing improves long-term immune outcomes require further study	(34)
Other solid tumors	Low immunogenicity and resistance to single cell-death pathways	HMME/Fe³^+^/tannic-acid ultrasound-activated HFT nanoplatform + anti-PD-1	Combined SDT and chemodynamic effects induced apoptosis, ferroptosis and ICD and increased T-cell infiltration	Relatively simple material integration compared with highly multicomponent platforms may facilitate mechanistic evaluation	Generalizability across tumor types, standardized acoustic dosing, PK/clearance, batch reproducibility and durable systemic immune protection remain to be demonstrated	(41)

## 9 Translational challenges and future perspectives

### 9.1 Safety, controllability and immune-related adverse events

Safety is one of the central translational challenges for ultrasound-responsive immunotherapeutic nanoplatforms. Conventional immune checkpoint blockade can produce immune-related adverse events involving the skin, gastrointestinal tract, endocrine organs, liver, lung, heart, and nervous system, which makes systemic immune activation a major clinical concern (112, 113). Although ultrasound-responsive systems are designed to restrict immune activation to the tumor region, this theoretical advantage still requires rigorous validation through pharmacokinetic studies, biodistribution analysis, long-term toxicity monitoring, and immune safety assessment (14, 16).

One key issue is that some sonosensitizers or immune payloads may have “always-on” activity before ultrasound exposure. Premature ROS generation, off-target drug leakage, or unintended immune agonist release could damage normal tissues or activate systemic inflammation (33, 42, 50). Activatable platforms address this problem by using ultrasound-cleavable linkers, ROS-responsive release, lipid oxidation, gas-generating modules, or tumor microenvironment-responsive chemistry to keep the platform relatively silent before local insonation. However, the field still needs standardized assays to compare pre-activation leakage, off-target immune activation, complement activation, hepatotoxicity, and repeated-dose safety across different materials (114).

Another safety consideration is the interaction between ultrasound dose and immune activation. Excessive acoustic intensity may cause nonspecific tissue injury, vascular damage, heating, or inflammatory toxicity, whereas insufficient ultrasound exposure may fail to activate the nanoplatform or induce ICD (103, 115). For immunotherapy, the goal is not maximal tumor destruction alone, but controlled immune activation that preserves antigen presentation and avoids destructive inflammation in normal tissues. Therefore, future studies should evaluate both antitumor efficacy and immune-related toxicity under repeated ultrasound exposure, especially for STING agonists, cytokine mRNA, and gas-generating platforms.

### 9.2 Experimental standardization, reproducibility, and robustness of immune evidence

A major barrier to cross-study comparison is the lack of standardized ultrasound parameters (115). Current studies vary widely in frequency, power density, acoustic pressure, duty cycle, pulse repetition frequency, exposure duration, treatment interval, probe geometry, focal depth, coupling method, and temperature monitoring (114, 115). These differences make it difficult to determine whether therapeutic outcomes are driven by material design, ultrasound dosing, tumor model differences, or immune assay variation (103, 115). Without standardized acoustic reporting, therapeutic outcomes are difficult to reproduce across laboratories or attribute specifically to nanoplatform design, because different acoustic regimens may shift the dominant mechanism among cavitation, sonoporation, heating, ROS generation, vascular permeabilization, and direct tissue injury. More importantly, this heterogeneity complicates mechanistic interpretation. A superior therapeutic outcome attributed to nanoplatform design may partly reflect differences in acoustic energy deposition, cavitation, sonoporation, vascular disruption, ROS generation, or heating. Cross-study comparisons should therefore be interpreted cautiously when acoustic exposure has not been adequately characterized or normalized.

Importantly, ultrasound modality should not be treated as interchangeable terminology because different exposure strategies favor distinct biophysical effects. Focused ultrasound (FUS) describes spatial concentration of acoustic energy and can be delivered across a range of intensities; therefore, its biological effects depend strongly on acoustic pressure, duty cycle, pulse structure, and exposure duration. Low-intensity pulsed ultrasound (LIPUS) generally emphasizes non-ablative mechanical effects and can facilitate cavitation, sonoporation, drug transport, and vascular permeability, particularly when combined with nanoparticles or cavitation nuclei. In contrast, high-intensity focused ultrasound (HIFU) deposits substantially greater acoustic energy and is more likely to produce thermal coagulation, mechanical disruption, and tissue ablation, although cavitation may also contribute depending on the exposure regime.

Microbubble-assisted insonation represents a distinct strategy because exogenous microbubbles lower the threshold for acoustic cavitation and can markedly enhance sonoporation and vascular permeability. This property is advantageous for localized drug, nucleic-acid, or immune-modulator delivery but may also increase endothelial injury, hemorrhage, or nonspecific tissue damage when acoustic exposure is excessive. ROS generation should likewise not be attributed to ultrasound intensity alone, because it depends on the presence and properties of sonosensitizers, cavitation behavior, oxygen availability, and acoustic exposure conditions. These modality-dependent effects are summarized in **Table 4**. At minimum, studies should report ultrasound frequency, acoustic intensity or pressure, duty cycle, pulse characteristics, exposure duration, focal geometry, coupling conditions, treatment interval, and temperature monitoring, with cavitation measurements included when cavitation is considered a major mechanism.

**Table 4:** Influence of ultrasound modality and exposure conditions on biophysical effects relevant to cancer immunotherapy.

Ultrasound modality	Defining exposure characteristics	Cavitation	Sonoporation	Heating	ROS generation	Vascular permeability	Tissue injury risk	Relevance to ultrasound-responsive immunotherapy	Key parameters requiring reporting	References
Focused ultrasound (FUS)	Spatial concentration of acoustic energy within a defined focal region; exposure may range from low-intensity pulsed to higher-energy regimens	Variable; strongly dependent on acoustic pressure, pulse structure, and presence of cavitation nuclei	Variable; enhanced when cavitation nuclei or microbubbles are present	Low to substantial depending on intensity and duty cycle	Context-dependent; enhanced in the presence of sonosensitizers and cavitation	Can be locally increased under appropriate mechanical exposure, particularly with microbubbles	Exposure-dependent; excessive acoustic pressure may cause vascular or tissue injury	Provides spatially confined activation and enables localized drug delivery, barrier modulation, or nanoplatform activation	Frequency; acoustic pressure/intensity; duty cycle; pulse duration; PRF; focal dimensions; exposure duration; temperature and/or cavitation monitoring	(119–121)
Low-intensity pulsed ultrasound (LIPUS)	Low-intensity, pulsed, generally non-ablative exposure; biological effects are strongly parameter- and sensitizer-dependent	Low–moderate; may become prominent in the presence of microbubbles, nanoparticles, or other cavitation nuclei	Prominent under appropriate conditions, particularly for drug/gene delivery	Generally limited compared with ablative ultrasound	Can be substantial during SDT when sonosensitizers and oxygen are available	May enhance microvascular and cellular permeability	Generally lower under controlled exposure, but cavitation-mediated vascular injury can occur	Suitable for non-ablative SDT, drug/gene delivery, sonoporation, and controlled immune modulation	Frequency; acoustic intensity/pressure; duty cycle; PRF; pulse duration; exposure time; sensitizer or cavitation-nuclei conditions	(122–124)
High-intensity focused ultrasound (HIFU)	High acoustic energy concentrated within a focal volume; commonly used for thermal or mechanical tissue ablation	Moderate–high depending on exposure regime; inertial cavitation can contribute to mechanical damage	May occur, but is usually not the principal therapeutic objective	Prominent; rapid temperature elevation can produce coagulative necrosis	May accompany cavitation and tissue stress, but is not necessarily the dominant mechanism	May be altered secondary to vascular disruption or ablation	Relatively high; includes thermal coagulation, vascular injury, and mechanical tissue disruption	Can generate tumor destruction and antigen release, but excessive ablation may limit controlled immune modulation and preservation of viable immune/stromal components	Frequency; acoustic power/intensity; focal geometry; sonication duration; duty cycle; peak temperature/thermal dose; cavitation monitoring where relevant	(125–127)
Microbubble-assisted insonation/UTMD	Ultrasound delivered in the presence of exogenous microbubbles; microbubble oscillation substantially lowers the threshold for cavitation	Prominent; stable or inertial cavitation depends on acoustic exposure	Prominent; transient membrane permeabilization is a major mechanism	Usually limited under appropriately controlled non-ablative conditions	Generally secondary/context-dependent rather than the principal mechanism	Prominent; transient endothelial and vascular-barrier permeabilization can enhance extravasation	Exposure-dependent; excessive inertial cavitation may cause endothelial injury, hemorrhage, or irreversible vascular damage	Particularly suited to localized delivery of drugs, nanoparticles, mRNA, genes, antibodies, and immune modulators	Frequency; peak negative pressure/mechanical index; pulse length; PRF; duty cycle; exposure duration; microbubble type/dose/size; injection-to-sonication timing; cavitation monitoring	(128–131)

The field also needs a unified immune-evaluation framework. In addition to tumor volume and survival, studies should include immune-cell profiling, antigen presentation markers, type I interferon signaling, checkpoint expression, memory T-cell responses, abscopal effects, and recurrence protection when possible. For studies focused on antitumor immune activation, orthotopic immunocompetent models are generally more informative than subcutaneous immunodeficient models because they preserve an intact host immune system and better recapitulate organ-specific tumor–immune interactions (116–118). This is particularly important for HCC, pancreatic cancer, GBM, and TNBC, where anatomical location and tissue-specific immune context strongly influence therapeutic response (96, 109).

### 9.3 Objective criteria for assessing translational potential

Because most ultrasound-responsive immunotherapeutic nanoplatforms remain at the preclinical stage, translational potential should not be inferred solely from therapeutic efficacy in murine models or from the novelty of platform design. In this review, translational potential is considered according to a defined set of criteria: (1) material biocompatibility; (2) biodegradability, biodistribution, and clearance; (3) manufacturing simplicity, scalability, and batch-to-batch reproducibility; (4) compatibility with clinically achievable and controllable ultrasound parameters; (5) pharmacokinetic behavior and tumor exposure; (6) short- and long-term safety, including repeated-dose and immune-related toxicity; (7) validation in orthotopic, immunocompetent, metastatic, or other clinically relevant models; and (8) reproducibility of therapeutic and immune effects across experimental systems. Platforms lacking data in these domains should therefore be regarded as promising preclinical concepts rather than clinically translatable technologies.

Clinical familiarity with individual platform components may reduce some translational barriers, but it should not be equated with readiness of the complete therapeutic system for clinical use. Microbubbles, lipid-based carriers, porphyrin derivatives, and related sonosensitizers benefit from existing experience in imaging, drug delivery, or biomedical manufacturing. However, incorporation of immune agonists, targeting ligands, responsive linkers, gas-generating modules, or ultrasound-triggered release mechanisms creates new pharmacokinetic, manufacturing, and safety requirements. Accordingly, prior clinical use of one component should be considered supportive evidence rather than proof of translational readiness of the final nanoplatform.

Several emerging strategies, including STING agonist delivery, mRNA immunotherapy, and gas-enhanced sono-immunotherapy, have shown encouraging preclinical activity but remain at different stages of translational evaluation. STING agonist platforms can turn local DNA damage and innate immune sensing into type I interferon-driven antitumor immunity, but they require precise spatial control to avoid systemic inflammation (49, 50). mRNA delivery platforms can locally express cytokines such as IL-12 or IL-15 and thereby program the tumor microenvironment without repeated systemic cytokine administration (59). NO- and SO_2_-releasing platforms add vascular, redox, and gas-mediated regulatory dimensions to SDT and checkpoint therapy, creating multi-modal strategies that may be particularly useful in hypoxic and poorly perfused tumors.

Several practical barriers must be solved before these systems can move beyond proof-of-concept. Batch-to-batch reproducibility, scalable synthesis, storage stability, sterilization, carrier degradation, immune recognition, organ accumulation, and clearance pathways must be defined for each nanoplatform (132). For ultrasound equipment, clinical translation will require standardized treatment planning, real-time image guidance, acoustic dosimetry, cavitation monitoring, and safety thresholds for repeated insonation (14, 103). For immunotherapy combinations, future studies must define rational sequencing, optimal timing between ultrasound and checkpoint blockade, biomarkers of response, and criteria for patient selection.

Ultimately, clinically translatable sono-immunotherapy will require convergence between material engineering, ultrasound physics, tumor immunology, and clinical oncology. Platforms may warrant further translational investigation when they combine relatively simple and reproducible manufacturing, favorable biodegradation and clearance profiles, clinically achievable ultrasound exposure, pharmacokinetic characterization, comprehensive safety assessment, and validation in orthotopic or immunocompetent models. Importantly, fulfillment of any single criterion should not be interpreted as evidence of overall translational readiness (114). Importantly, increasing functional complexity does not necessarily increase translational value. Each additional targeting ligand, responsive linker, immune agonist, gas-generating component, or coating step increases the burden of physicochemical characterization, batch-to-batch consistency, quality control, and regulatory validation. Translational value should therefore be considered as a multidimensional property rather than inferred from therapeutic efficacy or material familiarity alone. Rather than pursuing increasingly complex all-in-one particles, future development should prioritize platforms in which each module has a clear translational purpose: tumor accumulation, ultrasound activation, immune priming, checkpoint release, TME remodeling, or safety control (132). **Figure 5** summarizes how ultrasound-responsive immunotherapy can be matched to cancer type-specific barriers. It also highlights the key translational requirements that must be addressed before these platforms can move from proof-of-concept studies toward clinically applicable sono-immunotherapy.

**Figure 5:**
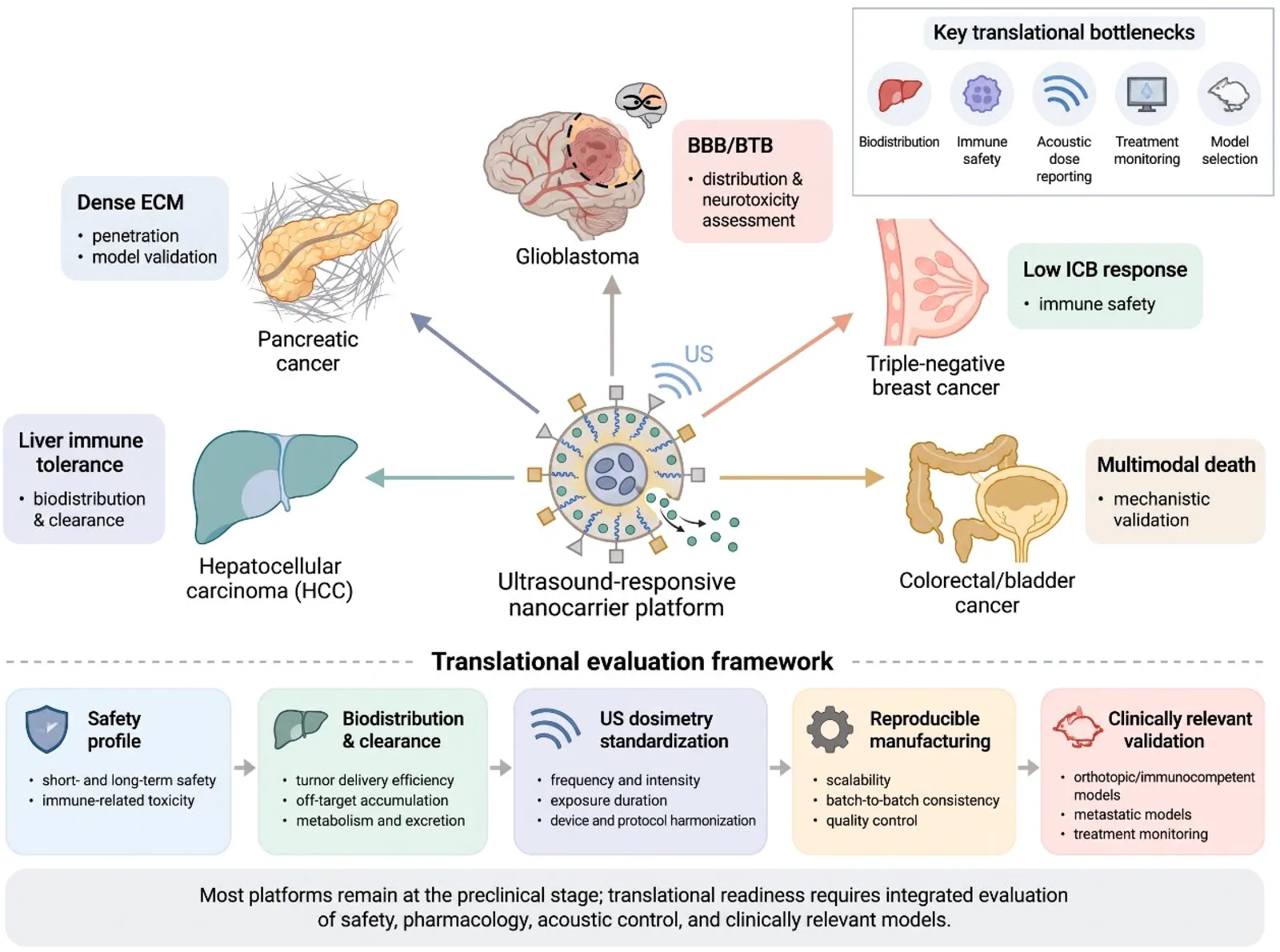
Cancer type-specific applications and translational roadmap. Ultrasound-responsive nanoplatforms can be tailored to distinct tumor barriers, including liver immune tolerance, pancreatic stromal density, BBB/BTB restriction, TNBC immune resistance, and multimodal cell-death requirements, while translation depends on safety, ultrasound dosimetry, manufacturing, and rational combinations.

## 10 Conclusion

Ultrasound-responsive nanoplatforms are shifting cancer immunotherapy from systemic and passively distributed immune modulation toward locally activated, temporally programmable, and microenvironment-adaptive immune engineering. By integrating sonodynamic ICD, innate immune activation, checkpoint blockade, mRNA delivery, regulated cell death, gas therapy, and tumor microenvironment remodeling, these platforms provide a versatile strategy to convert immune-cold tumors into immune-responsive lesions. Their greatest conceptual strength lies in the ability to align where immune activation occurs, when therapeutic payloads are released, and how the local tumor microenvironment is reshaped. Cancer type-specific applications further show that different tumors require different ultrasound-responsive designs. HCC may benefit from SDT-enhanced checkpoint blockade, pyroptosis, and SO_2_ gas therapy; pancreatic cancer requires deep activation, penetration enhancement, and stromal remodeling; GBM demands barrier-aware delivery and local immune programming; TNBC may be particularly suited for ultrasound-gated STING activation; and colorectal, bladder, and other solid tumors provide models for combining ICD with cytokine or cell-death pathway modulation. Future progress should therefore be judged not only by tumor-growth inhibition or increases in individual immune markers, but also by reproducibility across clinically relevant models, standardized acoustic exposure, causal evidence of immune dependence, functional demonstration of systemic and durable immunity, comprehensive long-term safety evaluation, and clinically compatible manufacturing.
